# Nectin-4 PET for predicting enfortumab vedotin dose-response in urothelial carcinoma

**DOI:** 10.1126/sciadv.ady1111

**Published:** 2026-01-07

**Authors:** Akhilesh Mishra, Ajay Kumar Sharma, Kuldeep Gupta, Dhanush S. Banka, Burles A. Johnson, Jean Hoffman-Censits, Peng Huang, David J. McConkey, Sridhar Nimmagadda

**Affiliations:** ^1^ The Russell H. Morgan Department of Radiology and Radiological Science, Johns Hopkins University School of Medicine, Baltimore, MD 21287, USA.; ^2^ The Johns Hopkins Greenberg Bladder Cancer Institute, Johns Hopkins University School of Medicine, Baltimore, MD, 21287, USA.; ^3^ Department of Oncology, The Sidney Kimmel Comprehensive Cancer center, Johns Hopkins University School of Medicine, Baltimore, MD, 21287, USA.; ^4^ Department of Biostatistics, Johns Hopkins University School of Medicine, Baltimore, MD, 21287, USA.; ^5^ Department of Pharmacology and Molecular Sciences, Johns Hopkins University School of Medicine, Baltimore, MD, 21287, USA.; ^6^ Division of Clinical Pharmacology, Department of Medicine, Johns Hopkins University School of Medicine, Baltimore, MD, 21287, USA.

## Abstract

Optimizing dosing strategies is critical to balance effectiveness and toxicity, especially for drugs with narrow therapeutic windows such as antibody-drug conjugates (ADCs). This study evaluates whether positron emission tomography (PET) imaging targeting Nectin-4 can noninvasively quantify the real-time interaction of the ADC enfortumab vedotin (EV) with tumors in urothelial carcinoma. Using the imaging agent [^68^Ga]AJ647, dynamic changes in the interaction of EV with Nectin-4 were measured across preclinical models and correlated with therapeutic responses. PET imaging identified dose-dependent variations in Nectin-4 engagement, with suboptimal EV doses resulting in incomplete Nectin-4 engagement and increased tumor growth. Crucially, PET-measured target engagement predicted therapeutic outcomes more reliably than either drug dose or baseline target expression. By defining effective target engagement levels needed for optimal therapeutic outcomes, PET imaging provides a clear benchmark for dosing decisions, maximizing efficacy while potentially reducing exposure to higher, toxic doses and thereby enhancing patient safety.

## Teaser

Real-time PET imaging of Nectin-4 interactions unlocks tailored dosing strategies for precision cancer therapy.

## INTRODUCTION

Determining optimal drug dosing is critical in drug development, directly affecting clinical efficacy and patient safety. Suboptimal dosing can lead to severe toxicities or therapeutic failures, reducing patient quality of life (*1*). Historically, dose-selection strategies have focused on identifying the maximum tolerated dose, an approach originating from cytotoxic chemotherapy (*2*). While effective for older therapies, this method is increasingly viewed as inadequate for modern targeted therapies, such as antibody-drug conjugates (ADCs), which have narrow therapeutic windows and complex mechanisms of toxicity (*1*, *3*).

In response, initiatives such as Project Optimus of Food and Drug Administration (FDA), alongside industry efforts, advocate shifting from identifying the highest tolerable dose toward establishing an optimal balance between efficacy, safety, and tolerability (*4*, *5*). However, current paradigms struggle to account for the complex interactions between drugs and their targets within the tumor microenvironment, presenting considerable challenges to pharmacodynamics (PD) biomarker discovery (*6*, *7*). To address these limitations, we proposed integrating advanced imaging biomarkers with conventional pharmacokinetics (PK) and PD assessments. Imaging, particularly through methods such as positron emission tomography (PET), offers a complementary layer of data that enhances traditional PK and PD assessments. While PK/PD data provide insights into drug distribution and activity over time, imaging captures real-time, spatially resolved measurements of drug distribution and drug-target interactions within the tumor and across the whole body (*8*). Unlike traditional plasma-based PK analyses that overlook tumor-specific drug uptake barriers, PET imaging directly quantifies target availability at tumor sites.

**Fig. 1.**
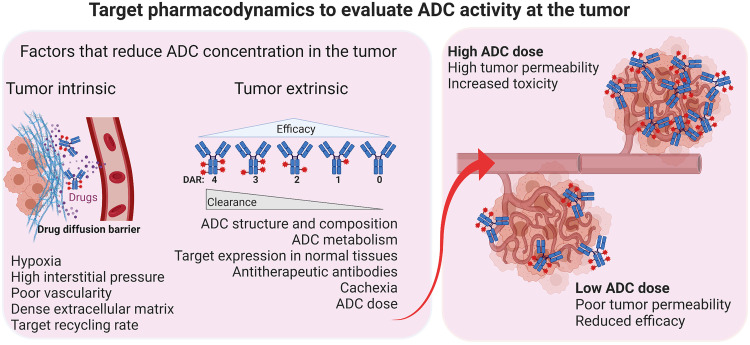
Mechanisms influencing ADC activity at the tumor site, highlighting tumor-intrinsic and tumor-extrinsic factors that reduce ADC concentration, efficacy, and DAR. Tumor-intrinsic barriers include hypoxia, high interstitial pressure, poor vascularity, dense extracellular matrix, and drug recycling rates, which impede ADC delivery and penetration. Tumor-extrinsic factors, such as ADC structure and composition, DAR variability, metabolism, off-target expression in normal tissues, antitherapeutic antibodies, and systemic conditions like cachexia, further affect ADC clearance and efficacy. The diagram contrasts outcomes of high ADC doses (enhanced tumor permeability but increased toxicity) with low ADC doses (reduced efficacy because of poor tumor permeability). These factors highlight the complex interplay of barriers that must be addressed to enhance ADC activity at the tumor site.

In this context, we hypothesized that PET imaging could enhance the understanding of ADC pharmacology, particularly regarding drug-target interactions within tumors. ADC efficacy is often limited by inadequate accumulation at tumor sites because of poor vascularization and high interstitial pressures, which conventional PK measurements fail to capture adequately (Fig. 1) (*9*). In addition, conventional PK and PD assessments often fall short in capturing the complexities of drug-target interactions within tumors, especially given the variability in target expression across different cancers and the poorly understood factors in the tumor microenvironment that influence drug accumulation (*6*, *8*). Our team designed a PET radiotracer ([^68^Ga]AJ647), a bicyclic peptide radiotracer targeting Nectin-4, specifically engineered with weaker affinity compared to enfortumab vedotin (EV) to ensure rapid clearance, enabling real-time quantification of target availability and drug-target interactions without saturating the receptor sites or perturbing EV pharmacology (*10*).

Focusing on EV, an FDA-approved ADC targeting Nectin-4 (*11*), we aimed to test whether PET-measured target engagement (TE) could better define the relationships among dose, exposure, and therapeutic response. Clinical trials indicated greater efficacy with higher EV doses but also increased dose adjustments and associated toxicities. In the EV-101 study, although responses were observed at all dose levels of EV, the 1.25 mg/kg dose level was associated with a greater tumor response, while lower response rates were observed at doses below 1.25 mg/kg (*12*). Adjustments in the dosage were more frequently necessary at the 1.25 mg/kg level, affecting more than 32% of patients compared to those on lower dosages resulting from toxicities associated with the higher doses of EV (*12*). These clinical findings indicate that understanding total Nectin-4 and engagement dynamics is paramount to understanding the response to EV treatments. Although previous Nectin-4–targeted imaging studies provided insights into tumor heterogeneity (*13*, *14*), they have not yet explored drug-target interactions or dose-response dynamics. Our approach addresses this critical knowledge gap by providing quantitative measurements of drug-target interactions early in treatment, potentially enabling precise and timely optimization of dosing strategies to improve ADC efficacy and reduce toxicity.

## RESULTS

### Synthesis and characterization of [^68^Ga]AJ647 as a Nectin-4 binding moiety

**Fig. 2.**
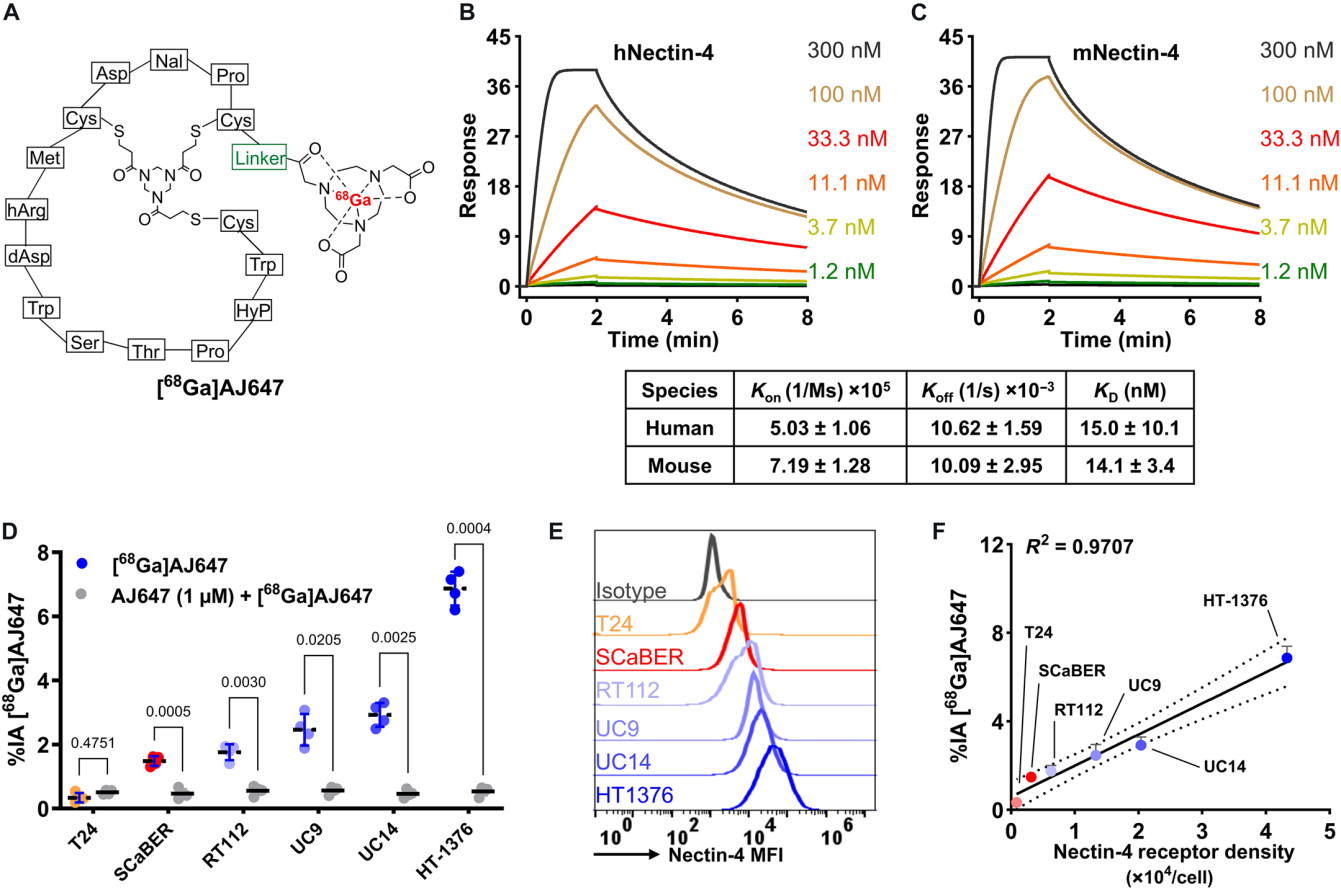
Structural design and chemical and in vitro specificity assessment of AJ647/[^68^Ga]AJ647. (**A**) Molecular structure of AJ647, a bicyclic peptide having NOTA as a bifunctional chelator for ^68^Ga labeling. (**B** and **C**) SPR analysis showing the binding affinity of AJ647 toward Nectin-4, assessed using recombinant human and mouse Nectin-4 proteins, respectively (*n* = 3). (**D**) [^68^Ga]AJ647 binding (%IA) to bladder cancer cell lines of basal (red) and luminal (blue) morphological phenotypes. Cells were incubated with ∼37-kBq (∼1 μCi) [^68^Ga]AJ647 at 4°C for 30 min, then excess unbound [^68^Ga]AJ647 was washed off, and bound activity was normalized against 1-μCi [^68^Ga]AJ647 standard tubes. [^68^Ga]AJ647 uptake is Nectin-4 expression–dependent, and co-incubation with 2 μM nonradioactive AJ647 (0.2 nmol, blocking dose) significantly reduced radiotracer uptake, confirming Nectin-4 specificity. (**E**) Flow cytometry analysis of Nectin-4 receptor expression across different bladder cancer cell lines stained with an anti–Nectin-4 antibody, providing insight into the variability in receptor density among the cell lines. A representative of three independent experiments is shown. (**F**) Correlation between in vitro [^68^Ga]AJ647 uptake and Nectin-4 receptor density, demonstrating that the uptake of [^68^Ga]AJ647 is dependent on the cell surface density of Nectin-4 receptors. Data in (B) to (D) and (F) are presented as the means ± SD (*n* = 4). Pearson correlation used in (F).

AJ647, derived from BT8009—a 15–amino acid bicyclic peptide containing a monomethyl auristatin E drug with low nanomolar affinity to Nectin-4 (*15*, *16*)—was modified for our purpose and further optimized to enhance its PK properties. A polyethylene glycol (PEG) linker was attached to the N-terminal cysteine residue to form AJ632 (Fig. 2A). The free terminal amine of AJ632 was then conjugated to a 1,4,7-triazacyclononane-1,4,7-triacetic acid (NOTA) chelator, enabling the successful radiolabeling with gallium-68 to produce [^68^Ga]AJ647 and its nonradioactive analog [^nat^Ga]AJ647. The radiolabeling process, performed as previously described (*17*), yielded [^68^Ga]AJ647 with a 71.2 ± 9.4% decay-corrected yield and a 97 ± 0.8% purity (*n* = 20), achieving high molar specific activity (figs. S1 to S5). The stability of [^68^Ga]AJ647 was confirmed for up to 3 hours in its final formulation (fig. S6). In addition, the compound exhibited a partition coefficient (log*D*) value of −2.06 ± 0.17, indicating high solubility in aqueous media (fig. S7). Surface plasmon resonance (SPR) studies demonstrated that AJ647 bound to human and mouse Nectin-4 with affinities of 15.0 ± 10.1 and 14.1 ± 3.4 nM, respectively (Fig. 2, B and C).

To assess the binding specificity of [^68^Ga]AJ647 to Nectin-4, we selected several urothelial carcinoma (UC) cell lines, including T24 (low Nectin-4, control cell line), SCaBER, RT112, UC9, UC14, and HT1376 (high Nectin-4). Upon incubation with [^68^Ga]AJ647, HT1376 cells demonstrated the highest uptake (6.5 ± 0.52), followed by UC14 (3.0 ± 0.42), UC9 (2.2 ± 0.48), RT112 (1.9 ± 0.18), SCaBER (1.6 ± 0.13), and T24 (0.2 ± 0.11), and co-incubation with an excess of nonradioactive AJ647 (0.2 nmol, blocking dose) significantly reduced radiotracer uptake, confirming Nectin-4 specificity (Fig. 2D). Flow cytometry analysis corroborated these results, showing a consistent pattern of Nectin-4 expression across these cell lines, with expression ranked as follows: T24 < SCaBER < RT112 < UC9 < UC14 < HT1376 (Fig. 2E). Furthermore, the quantification of cell surface Nectin-4 density using phycoerythrin (PE)–labeled beads revealed a strong correlation between receptor density and [^68^Ga]AJ647 uptake (Fig. 2F, *R*^2^ = 0.97). Collectively, these findings demonstrate the high in vitro selectivity of [^68^Ga]AJ647 for Nectin-4.

### PK of [^68^Ga]AJ647

**Fig. 3.**
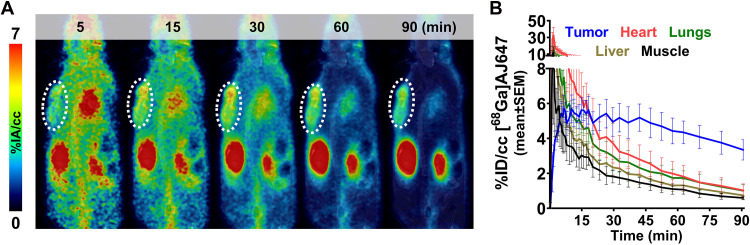
PK of [^68^Ga]AJ647 in NSG mice bearing HT1376 tumors. (**A**) Dynamic PET imaging of NSG mice injected with ∼9.25-MBq (∼250 μCi) [^68^Ga]AJ647. Images were acquired continuously from −1 to 90 min. The coronal section of fused PET-MR images at selected time points shows the accumulation and retention of [^68^Ga]AJ647 in the tumor (indicated by a white circle) and radiotracer’s washout from background tissues. (**B**) Time-activity curves of [^68^Ga]AJ647 uptake in the tumor, heart, lungs, liver, and muscle derived from dynamic scans from −1 to 90 min, showing the differential uptake and clearance of [^68^Ga]AJ647 in these tissues over time, highlighting its preferential retention in the tumor compared to other organs. %ID/cc, percentage injected dose per cubic centimeter. Data in (B) are presented as the means ± SEM (*n* = 3).

Given that HT1376 exhibited the highest Nectin-4 expression, we conducted a pharmacological evaluation of [^68^Ga]AJ647 in HT1376 xenografts using immunocompromised non-obese, diabetic, severe-combined immunodeficient gamma (NSG) mice. Dynamic PET-magnetic resonance (MR) imaging was performed for up to 90 min postinjection. PET images revealed significant radiotracer accumulation in HT1376 tumors as early as 15 min postinjection (Fig. 3A). By 60 min, the tumor contrast had further improved because of background radiotracer clearance, as evidenced by the percent injected activity per cubic centimeter (%IA/cc) values derived from [^68^Ga]AJ647 PET (Fig. 3B). The specificity of [^68^Ga]AJ647 for Nectin-4 was confirmed by the reduction in tracer uptake in HT1376 xenografts following administration of nonradioactive AJ647 (2 mg/kg; fig. S8). Notably, [^68^Ga]AJ647 uptake was also observed in the skin and salivary glands, consistent with the expression of Nectin-4 in these tissues (fig. S8). In contrast, minimal uptake was detected in non–Nectin-4–expressing tissues such as muscle.

To corroborate the PET imaging results, ex vivo biodistribution studies were performed, in which mice were injected and tracer uptake in tissues was quantified by harvesting the tumors and different organs after euthanizing the mice (Table 1). [^68^Ga]AJ647 uptake in Nectin-4–expressing tumors reached a maximum of 3.75 ± 0.86 %IA/g at 15 min, with similar retention observed at 60 min (3.72 ± 0.36). Blood pool activity demonstrated efficient clearance, with more than 90% of the activity cleared by 60 min. Comparable clearance patterns were observed in all other nonspecific tissues. Consequently, high tumor-to-muscle (10.07 ± 2.19) and tumor-to-blood (4.60 ± 1.07) ratios were achieved at 60 min. Among normal tissues, the kidneys and bladder showed the highest radiotracer accumulation, consistent with the renal clearance mechanism typical for low-molecular-weight peptides. On the basis of these findings, we determined that the 60-min time point provided optimal contrast and aligned well with standard PET clinical workflows, making it the preferred time point for subsequent experiments.

**Table 1.** PK of [^68^Ga]AJ647 in mice bearing HT1376 tumors. Mice bearing HT1376 tumors were injected with ∼1.85-MBq (∼50 μCi) [^68^Ga]AJ647 in 200 μl of saline containing 5% ethanol. Mice were euthanized at specified time points postinjection, and tissues were harvested for analysis (*n* = 5 per group). Values represented as the means ± SD.

%IA/g or ratio	Time (min)				
	2	15	60	120	180
Blood	35.75 ± 3.64	2.94 ± 0.68	0.84 ± 0.22	0.28 ± 0.06	0.32 ± 0.09
Skin	1.13 ± 0.16	2.74 ± 0.50	1.45 ± 0.33	0.82 ± 0.22	0.66 ± 0.12
Muscle	0.96 ± 0.19	0.93 ± 0.36	0.38 ± 0.06	0.20 ± 0.11	0.18 ± 0.05
Femur	1.88 ± 0.29	1.30 ± 0.05	0.57 ± 0.18	0.27 ± 0.09	0.34 ± 0.09
Salivary glands	6.30 ± 1.24	4.74 ± 0.94	1.48 ± 0.18	0.67 ± 0.09	0.69 ± 0.07
Tumor	0.72 ± 0.31	3.75 ± 0.86	3.72 ± 0.36	2.06 ± 0.49	1.39 ± 0.32
Lung	24.18 ± 7.05	3.66 ± 0.50	1.66 ± 0.25	0.81 ± 0.28	0.47 ± 0.20
Thymus	15.69 ± 5.42	2.38 ± 0.70	0.71 ± 0.30	0.67 ± 0.21	0.52 ± 0.23
Heart	11.34 ± 0.51	1.15 ± 0.34	0.43 ± 0.07	0.19 ± 0.01	0.22 ± 0.01
Liver	6.45 ± 1.39	1.19 ± 0.30	0.45 ± 0.09	0.21 ± 0.07	0.23 ± 0.02
Stomach	0.34 ± 0.11	0.49 ± 0.36	0.27 ± 0.23	0.13 ± 0.04	0.16 ± 0.07
Small intestine	0.85 ± 0.23	0.68 ± 0.23	0.52 ± 0.17	0.16 ± 0.02	0.28 ± 0.04
Pancreas	2.39 ± 0.72	2.50 ± 0.38	0.93 ± 0.03	0.30 ± 0.06	0.42 ± 0.02
Spleen	1.04 ± 0.23	4.32 ± 1.31	1.75 ± 0.21	0.84 ± 0.17	0.83 ± 0.07
Large intestine	0.25 ± 0.16	0.41 ± 0.11	0.25 ± 0.18	0.18 ± 0.03	0.18 ± 0.05
Adrenals	4.81 ± 2.58	2.60 ± 1.10	4.45 ± 2.03	3.11 ± 1.01	3.80 ± 0.47
Kidney	4.32 ± 1.31	20.43 ± 3.7	30.82 ± 4.37	17.33 ± 12.86	24.82 ± 4.78
Bladder	1.72 ± 0.24	24.13 ± 15.31	39.52 ± 21.31	22.60 ± 6.46	58.04 ± 24.76
Testicles	0.79 ± 0.07	0.86 ± 0.12	0.74 ± 0.20	0.52 ± 0.17	0.93 ± 0.50
Brain	0.54 ± 0.15	0.10 ± 0.02	0.06 ± 0.01	0.06 ± 0.02	0.06 ± 0.02
Tumor/blood	0.02 ± 0.01	1.41 ± 0.40	4.60 ± 0.47	7.27 ± 0.76	4.58 ± 1.33
Tumor/muscle	0.79 ± 0.45	4.23 ± 0.94	10.07 ± 2.19	11.26 ± 3.03	8.20 ± 4.02

### Evaluation of [^68^Ga]AJ647 specificity in UC xenografts

**Fig. 4.**
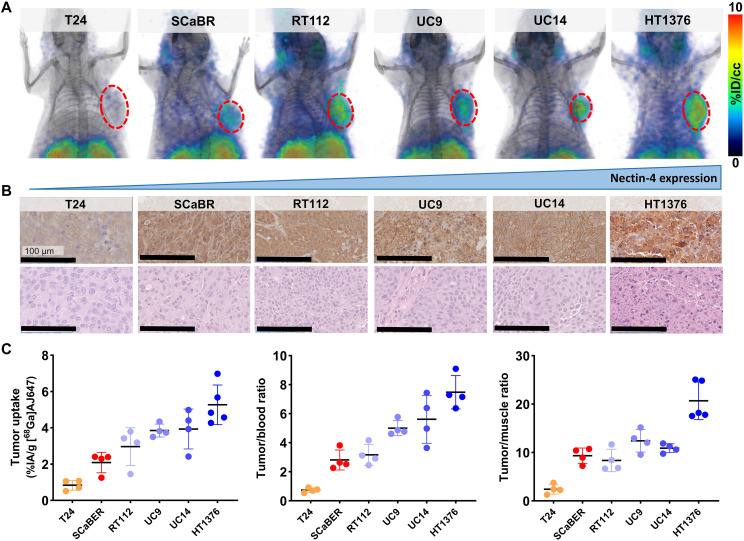
In vivo specificity of [^68^Ga]AJ647 for Nectin-4 in UC tumor xenografts. (**A**) Whole-body static PET-CT images acquired 60 min after intravenous injection of ~7.4-MBq (∼200 μCi) [^68^Ga]AJ647 in NSG mice bearing UC tumor xenografts (red: basal; blue: luminal phenotype). 3D volume-rendered PET images reveal significantly higher radiotracer accumulation in the tumors (highlighted by a red circle) compared to background tissues, demonstrating the specificity of [^68^Ga]AJ647 for Nectin-4. (**B**) IHC staining of Nectin-4 in corresponding tumor sections (imaged at a 20× resolution). Staining reveals variable Nectin-4 receptor densities across the tumor xenografts. (**C**) Tumor uptake and tumor-to-blood and tumor-to-muscle ratios derived from ex vivo biodistribution studies. NSG mice bearing UC xenografts were injected with ~740-kBq (∼20 μCi) [^68^Ga]AJ647 and euthanized 60 min postinjection. Ex vivo biodistribution data show the selective accumulation of [^68^Ga]AJ647 in tumors relative to blood and muscle. Data in (C) are presented as the means ± SD (*n* = 4 or 5).

To confirm the in vivo specificity of [^68^Ga]AJ647 for detecting varying levels of Nectin-4, we conducted studies using xenografts derived from the six UC cell lines previously used in our in vitro experiments. Consistent with our in vitro findings, the highest uptake of [^68^Ga]AJ647 was observed in HT1376 tumors, followed by UC14, UC9, RT112, and SCaBER, with the lowest uptake in T24 tumors (Fig. 4A). These findings were corroborated by immunohistochemical (IHC) staining of the same xenografts for Nectin-4 expression (Fig. 4B).

To validate these imaging findings, we performed ex vivo biodistribution studies. The results confirmed that the tumor uptake of [^68^Ga]AJ647 was consistent with both the in vitro data and PET imaging outcomes. In particular, the highest uptake was observed in HT1376 xenografts (%IA/g: 5.27 ± 1.09), followed by UC14 (3.93 ± 1.10), UC9 (3.85 ± 0.36), RT112 (2.96 ± 1.04), SCaBER (2.09 ± 0.56), and the lowest in T24 xenografts (0.84 ± 0.27), which corresponded well with their respective Nectin-4 expression observed in vitro by flow cytometry (Fig. 4C, left). In addition, tumor-to-blood (Fig. 4C, middle) and tumor-to-muscle (Fig. 4C, right) ratios mirrored these trends, further supporting the specificity of [^68^Ga]AJ647 for Nectin-4. Background tissues displayed no significant differences across the tumor models (fig. S9). Collectively, these results demonstrate that [^68^Ga]AJ647 is highly specific to Nectin-4 and effectively detects its varying expression in tumors in vivo.

### [^68^Ga]AJ647-PET could be used to quantify the Nectin-4 engagement of EV in vitro and in vivo

**Fig. 5.**
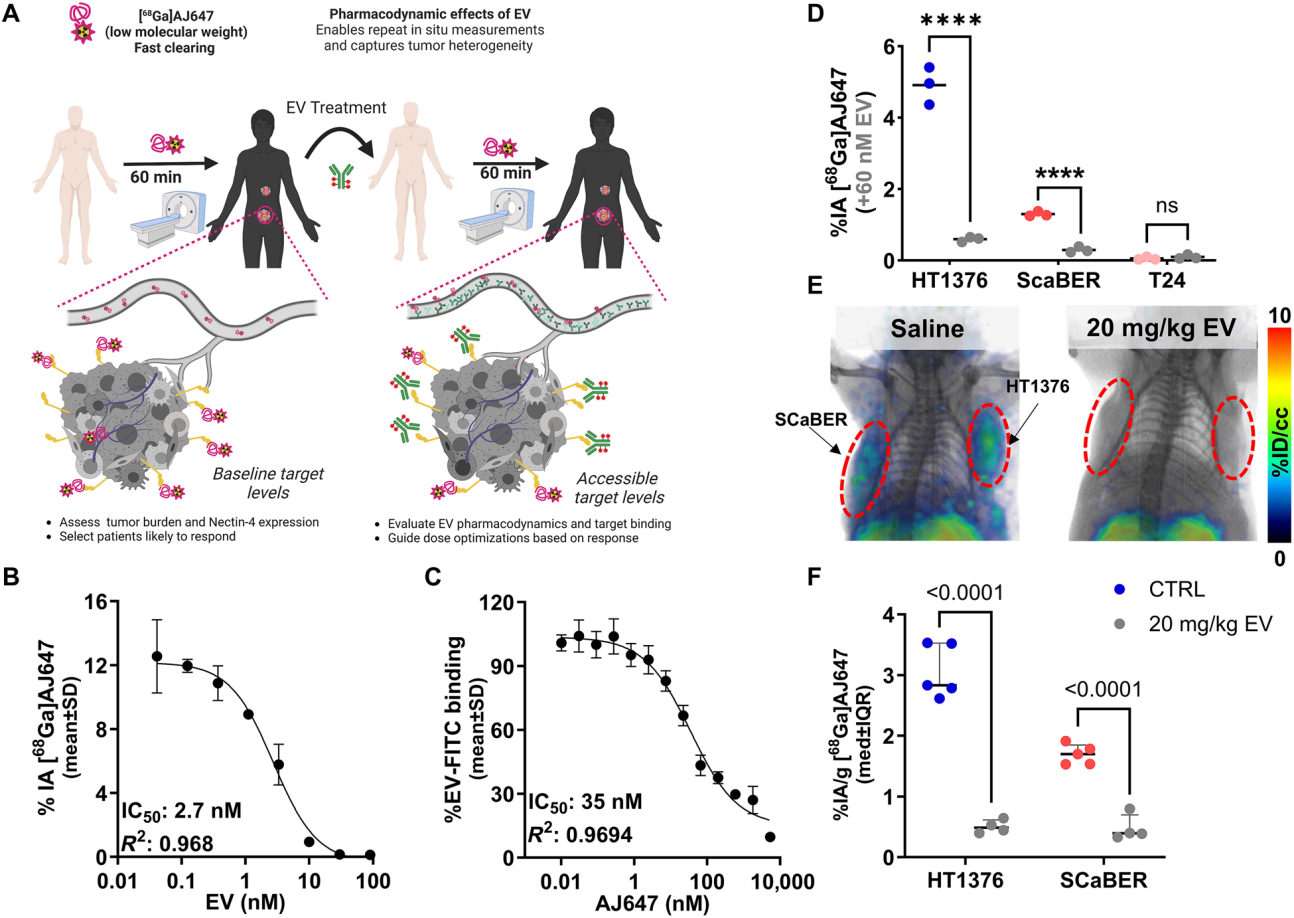
Competition binding assays of [^68^Ga]AJ647 and EV-FITC with Nectin-4 in the HT1376 cell line. (**A**) Schematic representation of competition assays between [^68^Ga]AJ647 and EV for binding to Nectin-4. On the left, the schematic illustrates the impact of varying EV concentrations on Nectin-4 levels, with accessible Nectin-4 quantified using [^68^Ga]AJ647. On the right, the schematic depicts how varying concentrations of AJ647 affect Nectin-4 levels, with accessible Nectin-4 determined by EV-FITC binding. (**B**) In vitro binding of [^68^Ga]AJ647 to HT1376 cells with varying concentrations of EV. The data show concentration-dependent inhibition of [^68^Ga]AJ647 binding, with an IC_50_ of 2.7 nM, indicating the competitive nature of EV in blocking [^68^Ga]AJ647 binding to Nectin-4. (**C**) In vitro binding of EV-FITC to HT1376 cells, assessed by flow cytometry, with varying concentrations of AJ647. The results demonstrate concentration-dependent inhibition of EV-FITC binding, with an IC_50_ of 35 nM, highlighting that AJ647 competes with EV-FITC for Nectin-4 binding. Data are presented as the means ± SD (*n* = 3 or 4). (**D**) In vitro binding of [^68^Ga]AJ647 to HT1376, SCaBER, and T24 cells with and without 60 nM EV. ns, not significant. ****P < 0.0001. (**E**) In vivo PET-CT imaging in mice harboring SCaBER (left) and HT1376 (right) tumors at a saturating dose of EV (20 mg/kg) or saline (*n* = 2). PET-CT was acquired at 60 min after 7.4-MBq (∼200 μCi) [^68^Ga]AJ647 injection. (**F**) Ex vivo biodistribution in mice harboring SCaBER (left) and HT1376 (right) tumors treated with EV (20 mg/kg) or saline control (*n* = 5). Mice were euthanized at 60 min after 1.85-MBq (∼50 μCi) [^68^Ga]AJ647 injection. IQR, interquartile range. Student’s *t* test performed in (D) and (F).

Previously, we have demonstrated that low-molecular-weight peptide–based imaging agents that overlap in binding mode with the therapeutic antibodies could be used to quantify the TE of those therapeutics by measuring unoccupied (accessible) target expression (*10*, *18*). To investigate whether [^68^Ga]AJ647 could be used similarly to investigate the TE of EV, we performed in vitro competition assays (Fig. 5A). Initially, HT1376 cells were incubated with [^68^Ga]AJ647 in the presence of increasing concentrations of EV. The results revealed a dose-dependent reduction in [^68^Ga]AJ647 binding, with a median inhibitory concentration (IC_50_) of 2.7 nM (Fig. 5B). We then performed a reverse competition assay using fluorescently labeled EV [EV-fluorescein isothiocyanate (FITC)] as a probe while varying concentrations of nonradioactive AJ647 were introduced. This led to a similar dose-dependent reduction in EV-FITC binding, with an IC_50_ of 35 nM (Fig. 5C). To validate that these observations are applicable across variable Nectin-4 expression, we preincubated HT1376, SCaBER, and T24 cells that exhibit variable Nectin-4 expression with EV for 30 min (60 nM) and then incubated them with [^68^Ga]AJ647. We observed that in all the cell lines tested, the presence of EV significantly reduced [^68^Ga]AJ647 binding in Nectin-4–positive HT1376 and SCaBER cells (*P* < 0.0001) but not in Nectin-4–negative T24 cells (Fig. 5D). These findings confirm that [^68^Ga]AJ647 and EV bind to the same Nectin-4 epitope. Notably, the weaker binding affinity (higher IC_50_) of AJ647 for Nectin-4, compared to EV, suggests that [^68^Ga]AJ647, typically used at picomolar-range doses, is unlikely to disrupt EV-Nectin-4 interactions but binds only to accessible Nectin-4.

To assess Nectin-4 engagement by EV in vivo in a noninvasive manner, we used two UC xenograft models, which exhibit varying Nectin-4 expression. These models were selected to investigate the ability of EV to target Nectin-4 across different tumor profiles, as Nectin-4 expression is heterogeneous in UC and serves as a key therapeutic target (*19*). NSG mice bearing HT1376 and SCaBER cell–derived xenografts, which represent high and moderate Nectin-4 expression, respectively, were treated with a single dose of EV (20 mg/kg) administered intravenously 24 hours before the injection of [^68^Ga]AJ647. PET images were acquired 1 hour postinjection of [^68^Ga]AJ647 in the HT1376 model (Fig. 5E). Mice treated with EV exhibited a significant reduction in [^68^Ga]AJ647 uptake in tumors compared to saline-treated controls, indicating successful Nectin-4 engagement by EV.

The PET imaging data were corroborated by ex vivo biodistribution analysis, which showed a significant reduction in [^68^Ga]AJ647 uptake in EV-treated tumors compared to controls: 84% reduction in HT1376 tumors (*P* < 0.0001) and 72% reduction in SCaBER tumors (*P* < 0.001) (Fig. 5F). These results demonstrate that [^68^Ga]AJ647 PET imaging can be effectively used to quantify in vivo Nectin-4 TE by EV, offering a valuable tool for monitoring drug-target interactions in tumors with varying Nectin-4 expression. The decrease in radiotracer accumulation was more pronounced in HT1376 tumors, correlating with their higher baseline Nectin-4 expression. These findings suggest a saturation of Nectin-4 by EV, particularly in tumors with greater receptor availability.

### Nectin-4 PET quantifies the dose-exposure relationship of EV at the tumor

ADCs, while designed to achieve a broad therapeutic window, often exhibit a relatively narrow margin compared to most monoclonal antibodies (*1*). This narrower therapeutic window frequently leads to dose reductions or early discontinuation of treatment as a result of toxicity concerns (*1*). A better understanding of dose-exposure dynamics, especially real-time monitoring of TE at the tumor, could enable more precise adjustments to dosing regimens and potentially expand the therapeutic window of ADCs.

**Fig. 6.**
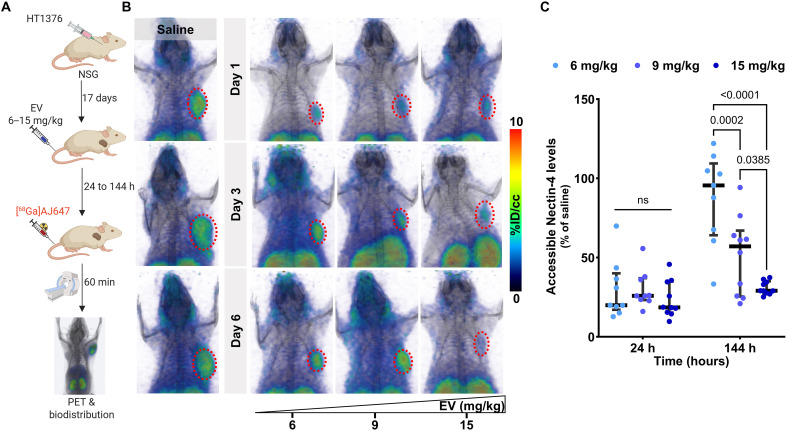
In vivo quantification of EV-blocked Nectin-4 using [^68^Ga]AJ647-PET in HT1376 tumor xenografts. (**A**) Schematic illustration of the experimental design showing how different doses of EV block Nectin-4 over various lengths of time. h, hours. (**B**) Whole-body static PET-CT images of saline (left; *n* = 2) or EV-treated mice (right; *n* = 3) at various doses and time points, illustrating the dose- and time-dependent blocking of Nectin-4 by EV. The mice received EV at FDA-approved human doses adjusted for mouse equivalents, administered with a single dose represented on the *x* axis. (**C**) Quantification of accessible Nectin-4 levels following EV treatment in tumor using ex vivo biodistribution at 1 day and 6 days posttreatment (*n* = 8 or 9 per dose per time point). Tumor uptake is expressed as the percentage to saline controls, providing a direct comparison of radiotracer accumulation with EV-naïve tumors. Refer to Materials and Methods for mathematical equations. A significant reduction in [^68^Ga]AJ647 binding was observed at 6 days, consistent with Nectin-4 blockade. Full biodistribution data are available in the Supplementary Materials. Data are presented as the median ± interquartile range. Two-way ANOVA used in (C).

In this study, we investigated whether Nectin-4 PET imaging could provide a quantitative assessment of changes in accessible Nectin-4 over time and track TE at the tumor in response to varying doses of EV. To evaluate this, we administered EV at doses of 6, 9, or 15 mg/kg, which are mouse equivalent doses (MEDs) corresponding to FDA-approved human doses of 0.5, 0.75, and 1.25 mg/kg, respectively, to mice bearing HT1376 tumors. Saline-treated mice served as controls (Fig. 6A). PET imaging with [^68^Ga]AJ647 was performed at 1, 3, and 6 days posttreatment to assess Nectin-4 engagement dynamics.

Saline-treated control mice exhibited a high uptake of [^68^Ga]AJ647, indicating the full availability of Nectin-4 receptors (Fig. 6B, left). On day 1, all EV doses effectively reduced [^68^Ga]AJ647 uptake in tumors, demonstrating the full occupancy of Nectin-4 across all dose levels (Fig. 6B, top). By day 3, however, an increase in [^68^Ga]AJ647 uptake was observed in the tumors of mice treated with the 6 mg/kg dose, suggesting that Nectin-4 became more accessible at this lower dose as the ADC cleared from the tumor. In contrast, tumors in the 9 and 15 mg/kg groups continued to show low tracer uptake, indicating sustained TE (Fig. 6B, middle). By day 6, the dose-dependent effects became even more pronounced. The 6 mg/kg group exhibited a significant increase in [^68^Ga]AJ647 uptake, reflecting the increased availability of Nectin-4, while the 9 mg/kg group showed a moderate increase in uptake, suggesting partial engagement. The 15 mg/kg group maintained minimal tracer uptake throughout, indicating the persistent high-level engagement of Nectin-4 by EV (Fig. 6B, bottom).

**Fig. 7.**
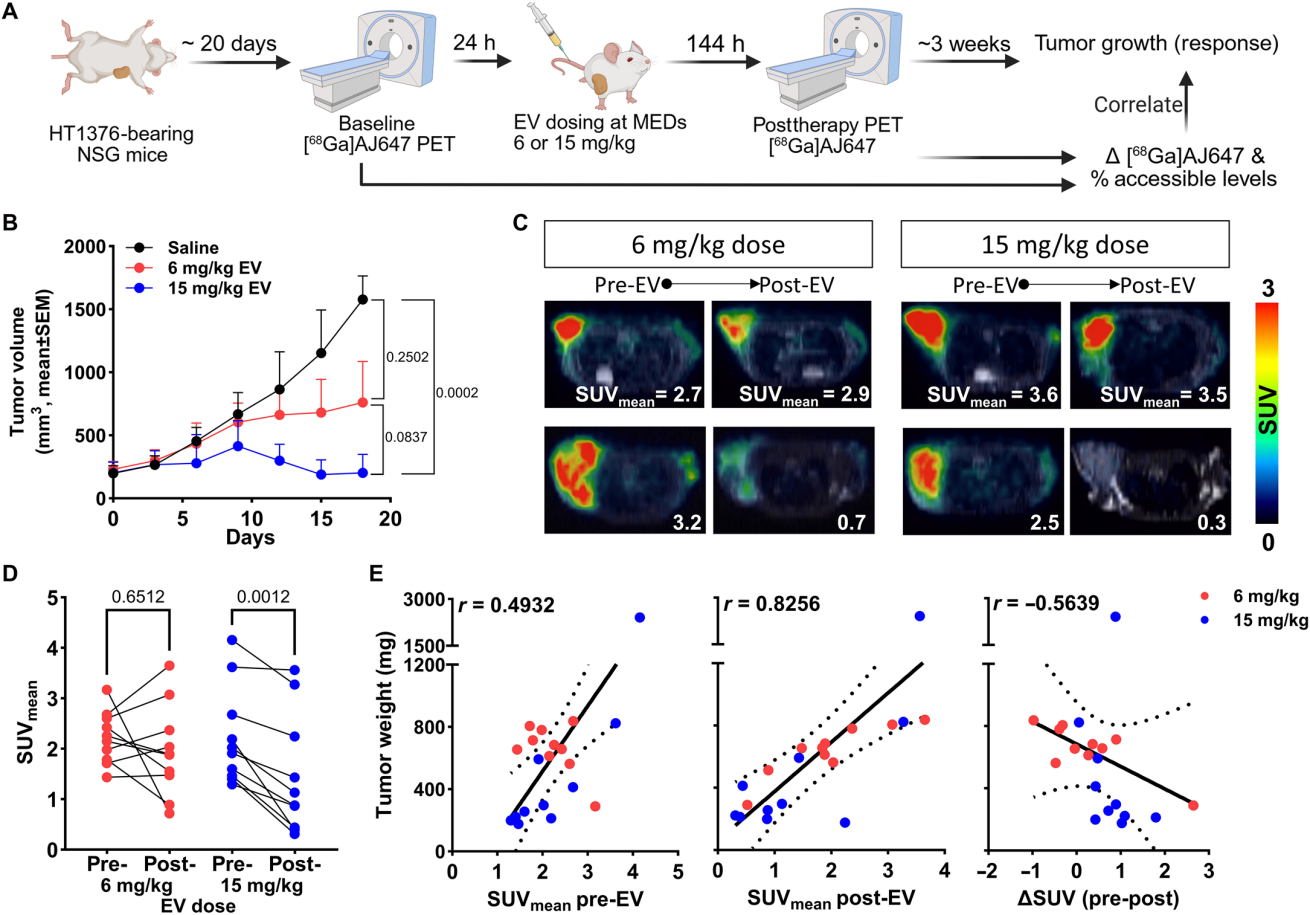
[^68^Ga]AJ647-derived Nectin-4 PD and correlation with response in the HT1376 tumor xenograft. (**A**) Schematic illustration of the experimental design showing the changes in the [^68^Ga]AJ647 tumor uptake following different doses of EV at day 6 [mouse equivalent doses (MEDs); the mice received EV at FDA-approved human doses adjusted for mouse equivalents, administered with a single dose]. (**B**) Tumor growth curve following a single dose (6 or 15 mg/kg) of EV treatment, showing growth inhibition in both EV-treated groups (*n* = 10 per group). (**C**) Fused trans-axial PET-MR images showing the differences in the SUVs of [^68^Ga]AJ647 pre– and post–6 days of EV treatment, indicating that EV can cause heterogeneous accessible Nectin-4 levels. (**D**) SUVs derived for each mouse from PET-MR imaging, showing the difference in uptake before and after EV treatment. (**E**) Correlation of tumor weights with tumor SUV before (left) and after (middle) EV treatment and difference (right) before and after EV treatment. Student’s *t* test used in (B) (unpaired) and (D) (paired). Spearman correlation used in (E).

Ex vivo biodistribution studies confirmed the PET imaging results by providing quantitative measurements of Nectin-4 engagement (shown as the percentage of saline control in Fig. 6C and %IA/g in fig. S10, A and B). These findings demonstrate that Nectin-4 PET imaging can effectively quantify the effects of dose and time on Nectin-4 engagement by EV, offering a valuable real-time tool for optimizing the dosing of EV and other Nectin-4–targeted therapeutics.

### Nectin-4 dynamics and correlation with the EV response

Further analysis of data in Fig. 7 revealed several important trends, including a dose-dependent decrease in tumor volumes at day 6 following EV treatment as well as notable variability in [^68^Ga]AJ647 uptake within tumors across different EV dose groups. Although we observed an inverse correlation between radiotracer accumulation and tumor weight (fig. S10C), the relationship was weak, suggesting that radiotracer uptake may reflect the tumor response to EV therapy, although other factors may contribute to this variability.

To investigate this further, we conducted experiments correlating EV dose, Nectin-4 PET measures, and response. Detailed experiment design is provided in fig. S11A. HT1376 tumor–bearing NSG mice were randomized and treated with single doses of EV (6 and 15 mg/kg), and tumor volumes were monitored for 2 weeks (fig. S11B). PET imaging using [^68^Ga]AJ647 was performed pretreatment (day 0) and posttreatment (day 6) (Fig. 7A). Tumor growth inhibition was observed in both groups but was more pronounced and consistent in the 15 mg/kg group (Fig. 7B). The 6 mg/kg group exhibited greater variability in tumor growth response, although both groups showed a dose-dependent response.

Analysis of PET images of those mice revealed the heterogeneous reduction in Nectin-4 PET signal after EV treatment in both dose groups (Fig. 7C). By day 6, the reduction in [^68^Ga]AJ647 uptake was more significant in the 15 mg/kg group, with 9 of 10 mice showing a drop in uptake compared to 5 of 10 in the 6 mg/kg group (Fig. 7D and fig. S11, C and D). This suggests a stronger therapeutic effect at the higher dose. However, further analysis was needed to determine which, if any, of the Nectin-4 PET measurements were the most relevant to predicting the therapeutic response. Correlation analysis showed that pretreatment PET measurements were only weakly associated with initial tumor volumes (*r* = 0.3203; fig. S11E) and final tumor weights (*r* = 0.4932; Fig. 7E, left). However, posttreatment standardized uptake values (SUVs) correlated more strongly with final tumor weights (*r* = 0.8256; Fig. 7E, middle), and the change in SUV from pre- to posttreatment (ΔSUV) was moderately negatively correlated with tumor weight (*r* = −0.5639; Fig. 7E, right). Both SUV_mean_ and SUV_max_ values showed similar trends (fig. S12).

### TE as a predictive biomarker for the EV therapy response

**Fig. 8.**
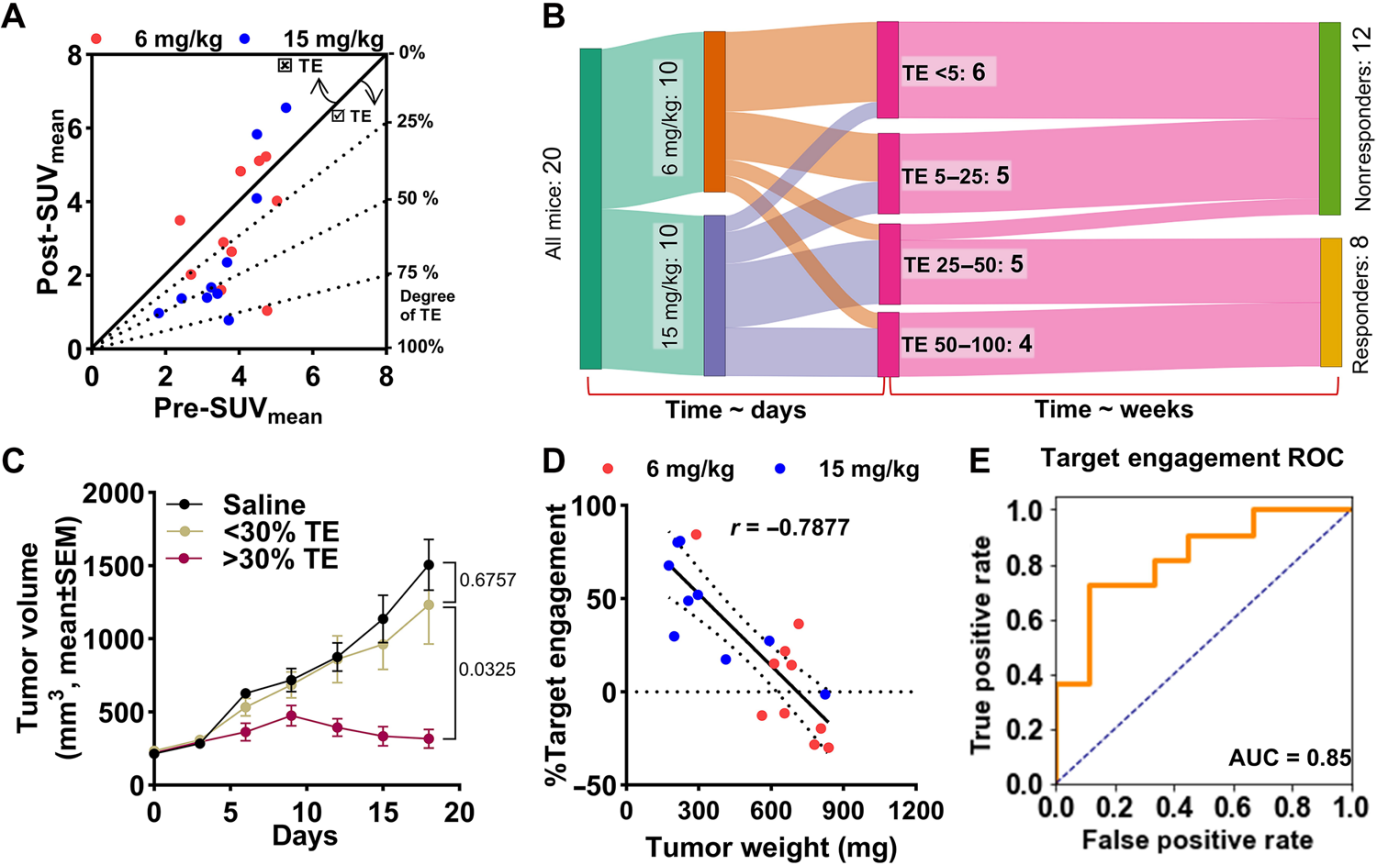
[^68^Ga]AJ647-derived EV TE with Nectin-4 and correlation with response. (**A**) Diagonal plot of posttreatment versus baseline SUV indicating the degree of EV TE. A higher degree of Nectin-4 engagement with EV is observed for points at a greater angle from the diagonal. Points above the diagonal line indicate no TE. (**B**) Sankey diagram illustrating the relationship between dose, TE, and response variability. The left side represents two dosing regimens, “6 mg/kg” and “15 mg/kg,” each with 10 samples. Data flow into four TE categories on the right: TE <5, TE 5 to 25, TE 25 to 50, and TE 50 to 100. The width of the connecting bands reflects the number of samples transitioning from each dose to a given TE category. A total of 11 samples exhibit low TE (TE <25) across both dosing regimens, with 6 samples in TE <5 and 5 samples in TE 5 to 25, across both dosing regimens, indicating variability in TE irrespective of dose. While a dose-dependent shift toward higher TE ranges (TE 25 to 50 and TE 50 to 100) is evident with the 15 mg/kg group, the persistence of low TE in some samples suggests potential biological or pharmacological factors influencing the response beyond dose. (**C**) Tumor growth curves based on Nectin-4 engagement showing the significant tumor growth inhibition in mice with at least 30% TE. (**D**) Correlation of tumor weights with TE. (**E**) The ROC curve shows that TE is a sensitive predictive measure of response. The mathematical equation of TE is provided in Materials and Methods. Pearson correlation used in (E).

Despite the insights gained from posttreatment SUVs, interpreting these values in isolation, without the knowledge of baseline Nectin-4 expression, poses challenges in clinical practice. This is because posttreatment SUVs can be influenced by other factors, such as baseline Nectin-4 expression variability or changes in the tumor microenvironment. Without a pretreatment baseline, assessing the full impact of EV treatment on TE becomes difficult, underscoring the importance of both pre- and posttreatment PET measurements for a comprehensive evaluation of therapeutic response. To integrate these measures into analysis, we derived the TE by dividing ΔSUV (SUV_post_ − SUV_pre_) with pretreatment SUV values (SUV_pre_). TE was also derived graphically by plotting SUV_post_ versus SUV_pre_, with the slope of the line from the origin representing the *y*/*x* ratio and indicating the extent of TE (Fig. 8A). We found a clear relationship between TE and tumor response and observed variable TE in both the 6 mg/kg and 9 mg/kg groups, with the higher dose group demonstrating greater TE.

Considering the variability observed in dose groups, we further sought to delineate the relationship between dose, TE, and response. A Sankey diagram was constructed to illustrate the relationship between dose, TE, and response (Fig. 8B). We defined responders on the basis of modified response evaluation criteria in solid tumors (mRECIST) for mice, distinguishing between response 0 (progressive disease) and response 1 (stable disease or better). The flow of data showed that both dose groups exhibited variable TE and mice with low TE led to a poor therapeutic outcome, regardless of the dose administered. Notably, nearly 33% of the mice showed less than 30% TE, while 50% exhibited less than 50% TE, highlighting a significant variability in response. Replotting the tumor volume data on the basis of TE revealed that mice with more than 30% TE exhibited significantly better responses to EV, regardless of dose (Fig. 8C). This replotting also resulted in a clearer separation of tumor growth curves compared to those plotted by dose alone. Moreover, a strong negative correlation was observed between TE and final tumor weight (*r* = −0.7877) (Fig. 8D), indicating that the level of TE is predictive of therapeutic outcome.

Further analysis explored the predictive value of PET imaging–derived biomarkers, particularly TE, for evaluating the therapeutic response. To quantitatively assess the predictive power of TE as a biomarker of response, we used receiver operating characteristic (ROC) curve analysis. ROC curves provide a statistical tool for evaluating the sensitivity (true positive rate) and specificity (true negative rate) of a biomarker across varying threshold levels, enabling the identification of the optimal cutoff for distinguishing between responders and nonresponders to therapy.

The ROC curves based on and pre- and posttreatment SUV values alone demonstrated poor predictive power, as indicated by the area under the curve (AUC) of less than 0.5, showing poor discriminatory power of using any parameter alone (fig. S13, A and B). The ROC curve derived from the pre/postchange had an area of 0.75, showing the predictability of response (fig. S13C). In contrast, the ROC curve derived from TE values (ΔSUV normalized to pretreatment SUVs) exhibited superior predictive accuracy with an AUC of 0.87 (Fig. 8E). This stronger predictive performance identifies TE as a more reliable classifier for assessing therapeutic outcomes compared to pre- and posttreatment SUVs. Moreover, the ROC analysis identified a ~30% TE threshold as a significant predictor of negative therapeutic outcomes (fig. S13D). Mice exhibiting less than 30% TE had significantly higher final tumor volume (759.4 ± 455 versus 324.9 ± 194.6; *P* = 0.0291) and were more likely not to respond to EV treatment (0 of 12 responders; 0%), while those exhibiting higher TE may derive benefit irrespective of dose they received (7 of 8 responders; 87.5%). This finding suggests that patients or tumor models showing low levels of TE are more likely to experience poor therapeutic outcomes and may require alternate dosing or treatment strategies.

Interpreting changes in tracer uptake after EV treatment requires careful consideration, as PET measures accessible target rather than the specific biological mechanism underlying its modulation. Reduced [^68^Ga]AJ647 uptake following EV treatment could arise from more than one mechanism, including antibody competition for Nectin-4 binding sites or payload-mediated cytotoxicity leading to a reduced accessible target. To address this, we reanalyzed our data focusing specifically on accessible Nectin-4 levels, which are what PET measures, rather than assuming a single underlying biological cause. These analyses confirmed that the accessible levels closely reiterate the findings from the TE analyses, and both approaches showed consistent relationships with tumor volume changes (fig. S14). These analyses support our interpretation that reduced tracer uptake reflects a reduced accessible target, whether as a result of receptor occupancy drug action or a combination of both. Nevertheless, comparison with the unconjugated antibody, particularly across multiple EV treatments, could provide further mechanistic resolution and represents a logical next step for future studies.

### Relationships between tumor outcomes, skin, and salivary gland uptake

Correlation analyses revealed that final tumor burden was most strongly explained by tumor PET-derived metrics (fig. S15). Tumor TE correlated inversely with tumor volume change (*r* = −0.84), identifying it as the dominant predictor of treatment response. Posttreatment tumor SUV also correlated with tumor volume (*r* = 0.76), although less strongly than TE. Uptake in physiologically Nectin-4–expressing tissues such as skin and salivary glands showed weaker and less consistent associations: Skin SUV_mean_ displayed a weak inverse correlation with tumor volume (*r* = −0.36), while skin TE and SG SUV_mean_ were only weakly positive (*r* = 0.27 and *r* = 0.38, respectively), and SG TE showed no meaningful relationship (*r* = −0.11). Normalization to tumor-to-skin and tumor-to-SG ratios improved predictive performance (*r* = 0.80 and *r* = 0.71, respectively), exceeding the strength of either tissue alone. Dose was moderately associated with outcome (*r* = −0.65) and inversely related to tumor TE (*r* = −0.48) but did not outperform PET-based predictors, and baseline tumor size was only weakly correlated with final volume (*r* = 0.34). Overall, these findings highlight tumor TE as the most robust and mechanistically relevant predictor of therapeutic response, outperforming SUV_mean_, baseline size, or dose. Uptake in skin and salivary glands alone was variable, and although skin SUV_mean_ showed a weak inverse association with tumor outcome consistent with a partial sink effect, this pattern was not reproduced across other measures. By contrast, normalization to tumor-to-normal tissue ratios restored predictive strength, supporting an exposure-surrogate framework in which uptake in physiological Nectin-4 tissues reflects systemic drug availability sufficient to engage both tumor and normal compartments.

## DISCUSSION

This study successfully demonstrates the utility of Nectin-4–targeted PET imaging as a noninvasive, real-time tool for optimizing dose-exposure-response relationships in EV therapy for UC. By leveraging the specificity of [^68^Ga]AJ647 for Nectin-4, we demonstrated the ability to dynamically quantify TE across a range of preclinical models, bridging critical gaps in current ADC development strategies and enabling a deeper understanding of drug-tumor interactions. We observed a strong correlation between PET-derived measures of TE and therapeutic outcomes. Mice exhibiting low Nectin-4 engagement posttreatment showed reduced tumor growth inhibition, regardless of dose, suggesting that TE itself may serve as a more reliable early biomarker of therapeutic efficacy than dose or Nectin-4 expression alone. These findings underscore the importance of integrating imaging-based biomarkers into drug development pipelines, particularly for complex therapeutics like ADCs, which have narrow therapeutic windows.

In vitro and in vivo competition studies confirmed that [^68^Ga]AJ647 binds specifically to the same epitope on Nectin-4 as EV, allowing for the accurate measurement of accessible Nectin-4. Our approach, using the bicyclic peptide tracer [^68^Ga]AJ647, offers significant practical advantages over traditional methods that directly radiolabel ADCs. Unlike labeled ADCs, which encounter the same barriers to tumor penetration as the therapeutic ADC itself, [^68^Ga]AJ647’s smaller size and rapid clearance enable the real-time measurement of accessible TE without disrupting ongoing therapy. Furthermore, because the imaging is completed within hours rather than the 5 to 7 days required for radiolabeled antibodies, PET-based measurements can be seamlessly integrated into routine clinical workflows, providing timely insights to inform dose adjustments and therapeutic decision-making. Our imaging approach quantified dose-dependent variations in TE, highlighting the consequences of suboptimal dosing, which led to incomplete engagement and reduced therapeutic efficacy. These insights are particularly valuable given the challenges of dose optimization in novel therapeutics. Since 2011, nine drugs receiving accelerated approval were later withdrawn primarily due to inadequate dosage optimization, resulting in unfavorable benefit-risk profiles (*20*). However, optimized dosing regimens allowed the reintroduction of two withdrawn drugs, including the ADC emtuzumab ozogamicin for CD33-positive acute myeloid leukemia (*5*). Integrating PET imaging into early clinical trials may similarly enhance dose optimization by providing complementary information on TE and dose-response dynamics.

This study also establishes TE as a predictive biomarker for therapeutic response. Using ΔSUV normalized to pretreatment values, we identified a strong correlation between TE levels and tumor growth inhibition. While our primary analyses focused on SUV_mean_, SUV_max_ showed comparable patterns. However, caution should be used when interpreting SUV_mean_ values if tumor volumes differ significantly between baseline and posttreatment time points. ROC analysis further identified a TE threshold of 32%, distinguishing responders from nonresponders and providing a quantitative benchmark for future studies. These findings align with emerging trends in ADC development, where integrating predictive biomarkers increasingly guide patient selection and therapeutic precision (*21*). For example, HER2 (human epidermal growth factor receptor 2)–directed ADCs rely on biomarker testing to identify HER2-overexpressing tumors, directly linking clinical response to target expression (*22*). In contrast, while Nectin-4 expression was initially used as a companion diagnostic for EV, substantial heterogeneity in its expression across metastatic UC has limited its predictive utility. Expression alone does not reliably differentiate responders from nonresponders, highlighting the necessity of assessing actual drug-target interactions within tumors. Recent evidence indicates that membranous Nectin-4 expression often declines during metastatic spread and correlates with EV response (*23*), although genomic amplification, strongly predictive of therapeutic outcomes, occurs in only a minority (26%) of EV-treated patients with metastatic UC (*24*). Thus, capturing dynamic TE through PET imaging may better address the variability in response and tumor heterogeneity, refining dosing strategies and therapeutic outcomes. In addition, early, noninvasive assessments of TE could support PK and PD evaluations during modifications to drug formulation, dosing schedules, or combination therapies, further enhancing treatment optimization.

Real-time assessment of tumor heterogeneity remains a critical challenge in optimizing targeted therapies. Despite the importance of sensitive assays for real-time insights into drug-tumor interactions, such tools remain limited (*6*, *25*). Model-based approaches that integrate nonclinical and clinical data—such as PK and PD parameters—can facilitate efficacy and safety predictions (*26*). However, these models often fail to fully account for the dynamic complexities of tumor heterogeneity and intrinsic factors that affect drug PK/PD, especially in real time. This is particularly true for macromolecular drugs like ADCs, where systemic exposure measurements may not adequately reflect tumor-specific drug distribution. The clinical relevance of our findings is further emphasized by comparisons to existing systemic exposure data from EV trials. In the EV-301 and EV-201 trials, the average systemic exposure (*C*_avg_) was not a statistically significant predictor of overall survival or best overall response, although trends indicated that higher exposure quartiles correlated with response rates of 55 to 64% compared to 32% in the lowest quartile (*27*, *28*). These findings suggest that blood-based exposure measurements may not accurately reflect tumor-specific drug behavior. By contrast, PET imaging provided direct, tumor-specific insights into Nectin-4 engagement, enabling a more precise understanding of dose-exposure-response relationships.

Nectin-4–targeted imaging also addresses the challenge of tumor heterogeneity in UC, where variable Nectin-4 expression affects therapeutic outcomes. While baseline Nectin-4 expression has a prognostic value, our data indicate that PET-derived engagement measures are more robust predictors of response. Longitudinal imaging studies could monitor dynamic changes in engagement, particularly during combination therapies or disease progression, enabling adaptive dosing strategies. In addition, PET imaging could be used to optimize dosing in earlier-stage UC or in patient populations with heterogeneous tumor profiles, expanding the therapeutic potential of EV.

Despite the promising predictive value of PET-derived TE, several limitations remain. First, while PET robustly measures antibody delivery and target binding, the contribution of the cytotoxic payloads to therapeutic outcomes is difficult to isolate. ADC efficacy and safety hinge not only on TE but also on payload dynamics, particularly considering time-dependent declines in drug-to-antibody ratio. Nonetheless, ADC exposure still correlates well with efficacy and safety end points in clinical contexts, suggesting that its variability may have a limited impact on overall outcomes (*28*–*31*). Second, our approach does not account for factors such as early tumor cell killing that could also contribute to the observed decline in tracer binding. While we anticipate tumor cell killing to be a minor contributor to the decline observed on day 1, further mechanistic studies using Nectin-4–independent methods, such as histological assessment and flow cytometry, will be valuable to distinguish drug-target engagement from tumor cell viability effects. In addition, while our PET approach offers advantages over traditional zirconium-89–labeled ADC methods—primarily improved real-time target measurement—its capacity to directly predict clinical toxicity remains untested and warrants further exploration. Future studies should focus on integrating PET-derived lesion–specific TE data with intracellular drug activity and conjugated payload measurements to better model therapeutic efficacy.

In summary, this study provides compelling evidence that the early assessment of drug-target interactions in real time with PET serves as a valuable tool for optimizing ADC therapy. By quantifying real-time TE, it addresses key limitations of conventional dose-finding paradigms and offers a pathway to enhance therapeutic precision. As ADCs and other targeted therapies continue to evolve, integrating imaging measures, like Nectin-4 PET for EV, could redefine dose selection strategies, improve patient outcomes, and accelerate the development of next-generation cancer treatments.

## MATERIALS AND METHODS

### Chemicals

NOTA-NHS ester was purchased from CheMatech, and tris(2-carboxyethyl)phosphine, triisopropyl silane, hydroxybenzotriazole, and hexafluorophosphate benzotriazole tetramethyl uronium were obtained from Chem-impex. All Fmoc-protected amino acids and Fmoc-PEG_12_-COOH were purchased from Ambeed, while *N*,*N*-diisopropylethylamine (DIPEA), trifluoroacetic acid (TFA), 3,6-dioxa-1,8-octanedithiol, 1,3,5-triacryloylhexahydro-1,3,5-triazine (TATA), and *N*,*N*′-dimethylformamide (DMF) were obtained from Sigma-Aldrich. All other chemicals were purchased from Sigma-Aldrich or Thermo Fisher Scientific.

### Cell culture reagents and antibodies

All cell culture reagents were purchased from Invitrogen (Grand Island, NY). EV (Nectin-4 ADC) was purchased from Johns Hopkins School of Medicine Pharmacy and used as is.

### Synthesis of AJ632

AJ632 was chemically synthesized using a Liberty Blue CEM automatic peptide synthesizer using Fmoc-based solid-phase peptide synthesis on Rink Amide resin in a 0.1-mmol scale (fig. S1). Coupling reaction was carried out using Oxyma (0.5 mmol), *N*,*N*′-diisopropylcarbodiimide (1 mmol), and Fmoc-AA-OH (0.5 mmol) in DMF with microwave-assisted reaction for 2 min. The Fmoc group was deprotected using 20% piperidine in DMF (3 ml) for 1 min with microwave assistance. Next, the PEG linker was incorporated by using hexafluorophosphate benzotriazole tetramethyl uronium (0.5 mmol), hydroxybenzotriazole (0.5 mmol), DIPEA (0.5 mmol), and Fmoc-NH-PEG_12_-COOH (0.5 mmol) in DMF at room temperature for 1.5 hours, and then the Fmoc group was deprotected using 20% piperidine (4 ml) in DMF for 40 min at room temperature. Once the sequence was completed on the resin, the peptidyl-resin was treated with 4 ml of cleavage cocktail (TFA:triisopropyl silane:3,6-dioxa-1,8-octanedithiol:H_2_O; 92.5:2.5:2.5:2.5) for 4 hours at room temperature. The cleaved reaction mixture was precipitated with diethyl ether to obtain the linear peptide as a white solid. For the cyclization, the linear peptide (102 mg, 0.043 mmol) was dissolved in 100 ml of water:acetonitrile (1:1) and treated with an aqueous solution of tris(2-carboxyethyl)phosphine (105 mg, 0.37 mmol) followed by Et_3_N (400 μl, 2.9 mmol). TATA (30 mg, 0.12 mmol) was dissolved in 2 ml of acetonitrile and added slowly to the reaction mixture over an hour. The reaction mixture was stirred at room temperature for 48 hours and quenched with TFA. The volatiles were removed, and the crude mixture was purified on a reversed-phase high-performance liquid chromatography (RP-HPLC) system using a preparative C-18 Phenomenex column (5 mm, 21.5 by 250 mm, Phenomenex, Torrance, CA). The HPLC condition was gradient elution starting with 5% acetonitrile:water (0.1% TFA) and reaching 50% acetonitrile:water (0.1% TFA) in 30 min, followed by isocratic run of 50% acetonitrile:water (0.1% TFA) until 40 min at a flow rate of 8 ml/min. The product AJ632 was collected at a retention time (RT) of ~30.9 min. Acetonitrile was evaporated under reduced pressure and lyophilized to form an off-white powder with a 25% yield (30 mg). The sequence of the AJ632 peptide is NH_2_-PEG_12_-(Cys-Pro-Nal-Asp-Cys-Met-hArg-dAsp-Trp-Ser-Thr-Pro-Hyp-Trp-Cys)-TATA–based cyclization. This peptide was characterized by matrix-assisted laser desorption/ionization–time-of-flight mass spectrometry (MALDI-TOF-MS). The theoretical chemical formula is C_125_H_183_N_25_O_38_S_4_ with an exact mass of 2770.2 and a molecular weight of 2772.2. The theoretical MALDI-TOF-MS mass [M + H]^+^ was 2771.2; the observed MALDI-TOF-MS [M + H]^+^ was 2773.9.

### Synthesis of AJ647

To a stirred solution of AJ632 (3.3 mg, 1.2 μmol) in 200 μl of DMF in a reaction vial, NOTA-NHS ester (2.5 mg, 2.8 μmol) and DIPEA (5 μl, 25 μmol) were added and stirred at room temperature for 4 hours (fig. S2). DMF was evaporated using a rotary evaporator under reduced pressure, and the residual product was purified on an RP-HPLC system using a semipreparative C-18 Luna column (5 mm, 21.5 by 250 mm, Phenomenex, Torrance, CA). The HPLC condition was gradient elution starting with 5% acetonitrile:water (0.1% TFA) and reaching 50% acetonitrile:water (0.1% TFA) in 30 min, followed by isocratic run of 50% acetonitrile:water (0.1% TFA) until 40 min at a flow rate of 8 ml/min. The product AJ647 was collected at RT ~31.2 min. Acetonitrile was evaporated under reduced pressure and lyophilized to form an off-white powder with a 27% yield, which was characterized by MALDI-TOF-MS. The theoretical chemical formula of AJ647 is C_137_H_202_N_28_O_43_S_4_ with an exact mass of 3055.3 and a molecular weight of 3057.5. The theoretical MALDI-TOF-MS mass [M + H]^+^ is 3056.3, and the observed ESI-MS mass [M + H]^+^ is 3058.9.

### Affinity measurements by SPR

The affinity of AJ647 for human and mouse Nectin-4 recombinant proteins was evaluated by SPR. The experiments were conducted using a Biacore T200 instrument with a CM5 chip at 25°C. The ligands used were His-tagged human Nectin-4 (R&D Systems, catalog no. 2659-N4-050, 44 kDa, 0.2 mg/ml stock concentration) and mouse Nectin-4 proteins (R&D Systems, catalog no. 3116-N4-050, 45 kDa, 0.2 mg/ml stock concentration), which were immobilized onto the CM5 chip. AJ647 (3057.5 Da, 10 mM stock concentration) was used as the analyte, which flowed over the ligand-immobilized surface. FC2 and FC4 was used as the experimental flow cell, while FC1 and FC3 served as the reference. An anti-His antibody (1 mg/ml stock concentration) was immobilized on all flow cells using standard amine coupling chemistry. The immobilization running buffer used was PBS-P [20 mM phosphate buffer, pH 7.4, 137 mM NaCl, 2.7 mM KCl, and 0.05% (v/v) surfactant P20]. Human Nectin-4 was captured onto FC2 at a level of ~600 RU, with a 1:20 dilution and 10 μg/ml diluted concentration in PBS-P. Mouse Nectin-4 was captured onto FC4 at a level of 600 RU, with a 1:20 dilution and 10 μg/ml diluted concentration in PBS-P. The theoretical maximum response (*R*_max_ ) values were calculated on the basis of the captured response values (table S1), assuming a 1:1 interaction mechanism. Overnight kinetics were performed for all analytes in the presence of PBS-P + 1% dimethyl sulfoxide. The flow rate of all analyte solutions was maintained at 50 μl/min. The contact and dissociation times used were 120 and 360 s, respectively. Surface regeneration was achieved by injecting glycine (pH 1.5) for 20 s, which takes away all captured ligands onto FC2 and FC4. Fresh ligands were captured at the beginning of each injection cycle. The analyte concentrations injected ranged from 300 nM down to 1.2 nM with threefold serial dilutions, and all analytes were injected in duplicate. All association and dissociation kinetics were evaluated by 1:1 kinetics model fitting.

### Synthesis of [^nat^Ga]AJ647

The synthesis of [^nat^Ga]AJ647 was carried out by adding 10 μl of aqueous 0.1 M [^nat^Ga]GaCl_3_ solution and 0.6 ml of 0.1 M HCl to a stirred solution of AJ647 (0.1 mg, 0.05 μmol) in 200 μl of 1 M NaOAc buffer (pH 5.0) in a reaction vial. The reaction mixture was incubated at 65°C for 30 min and then purified on an RP-HPLC system using a semipreparative C-18 Luna column (5 mm, 10 by 250 mm, Phenomenex, Torrance, CA). The HPLC condition was gradient elution starting with 20% acetonitrile:water (0.1% formic acid) and reaching 60% acetonitrile:water (0.1% formic acid) in 20 min at a flow rate of 5 ml/min. The product [^nat^Ga]AJ647 was collected at RT ~8.8 min. Acetonitrile was evaporated under reduced pressure and lyophilized to form an off-white powder, which was characterized by MALDI-TOF-MS. The theoretical chemical formula is C_137_H_200_GaN_28_O_43_S_4_ with an exact mass of 3122.2 and a molecular weight of 3125.2. The theoretical MALDI-TOF-MS mass [M + H]^+^ was 3123.3, and the observed ESI-MS mass [M + H]^+^ was 3124.5.

### [^68^Ga]AJ647 radiopharmaceutical preparation

The ^68^Ge/^68^Ga generator was manually eluted using 6 ml of 0.1 M HCl (ultrapure trace metal–free) in four different fractions (2.4, 1, 1, and 1.4 ml). To a microcentrifuge vial (1.5 ml) containing 200 μl of 1 M NaOAc buffer (pH 5) and 40 μg of AJ647 (13 nmol), 4 to 6 mCi of [^68^Ga]GaCl_3_ at 0.6 ml from the second fraction was added (fig. S4). The reaction mixture was incubated for 10 min at 65°C in a temperature-controlled heating block and purified on an RP-HPLC system using a semipreparative C-18 Luna column (5 mm, 10 by 250 mm, Phenomenex, Torrance, CA). The HPLC condition was gradient elution starting with 20% acetonitrile:water (0.1% formic acid) and reaching 60% acetonitrile:water (0.1% formic acid) in 20 min at a flow rate of 5 ml/min. The radiolabeled product [^68^Ga]AJ647 was collected at RT ~8.8 min, with a decay-corrected radiochemical yield of 71.2 ± 9.4% (*n* = 20). The desired radiolabeled fraction was concentrated under a stream of N_2_ at 60°C, formulated in 10% EtOH in saline, and used for in vitro and in vivo studies. The whole radiolabeling process was completed in ~30 min. Quality control, stability studies, and chemical identity were also performed on the same HPLC system using the same HPLC gradient as described above.

### Determination of the partition coefficient

The log*D* (pH 7.4) value was determined according to a literature procedure (*32*). Briefly, a 10-μl solution of [^68^Ga]AJ647 (around 10 μCi) was added to a solution of 1-octanol (200 μl) mixed with phosphate-buffered saline (PBS) (190 μl) in a 1.5-ml centrifuge tube. After the mixture was vigorously shaken and vortexed, it was centrifuged at 3000 rpm for 5 min. Aliquots (10 μl) were removed from both phases, and the radioactivity was measured on an automated gamma counter (1282 Compugamma CS, Pharmacia/LKB Nuclear Inc., Gaithersburg, MD). The log*D* was calculated as the average log ratio value of the radioactivity in the 1-octanol fraction and the PBS fraction from the three samples.

### Cell culture

All cell lines were purchased from American Type Culture Collection and cultured in the recommended media in an incubator at 37°C in a humid atmosphere containing 5% CO_2_. UC9, UC14, and RT112 were maintained in minimum essential medium. BFTC909 and SCaBER were maintained in Dulbecco’s modified Eagle’s medium. T24 cells were maintained in McCoy’s 5A medium. All cells were supplemented with 10% fetal bovine serum and 1% penicillin-streptomycin. All cell lines were authenticated using short tandem repeat profiling at the Johns Hopkins Genetic Core Facility. All cell lines were routinely tested for mycoplasma, and all experiments were performed within 10 to 12 passages after thawing.

### Detection of Nectin-4 expression by flow cytometry

Adherent cells were detached using enzyme-free cell dissociation buffer (Thermo Fisher Scientific, Waltham, MA). Nectin-4 surface expression was evaluated by direct staining of 1 × 10^6^ cells in 100 μl of fluorescence-activated cell sorting buffer (PBS with 0.1% fetal bovine serum and 2 mM ethylenediaminetetraacetic acid) with a PE-labeled anti–Nectin-4 antibody (clone: 337516, R&D, no. FAB2659P) for 30 min at 4°C. Cells were then washed and analyzed for the mean fluorescence intensity (MFI) by flow cytometry.

### In vitro binding assays with [^68^Ga]AJ647

In vitro binding of [^68^Ga]AJ647 to T24, SCaBER, RT112, UC9, UC14, and HT1376 cells was determined by incubating 1 × 10^6^ cells with ~1 μCi of [^68^Ga]AJ647 for 30 min at 4°C. After incubation, cells were washed three times with ice-cold PBS containing 0.1% Tween 20 and counted on an automated gamma counter. All cell radioactivity uptake studies were performed in quadruplicate for each cell line and repeated three times.

### Receptor density measurements

The PE Fluorescence Quantitation Kit (BD Biosciences, no. 340495) containing four levels of PE/beads were used. Beads were reconstituted, with 0.5 ml of PBS containing sodium azide and 0.5% bovine serum albumin, just before use. Cells were stained for 30 min at 4°C with a PE-labeled anti–Nectin-4 antibody (clone: 337516, R&D, no. FAB2659P) and run along with the beads to estimate receptor density using flow cytometry. The calibration curve of geometric mean versus PE/bead from different bead populations was generated as per the manufacturer’s protocol. This calibration curve was used to derive receptors/cell for each cell type from their respective geometric means. Isotype controls were used to eliminate any nonspecific staining. The Nectin-4 receptor density measured above was then correlated with [^68^Ga]AJ647 %IA uptake measured in in vitro binding assays.

### Mouse strains and in vivo studies

Xenografts were established in 5- to 6-week-old, male NSG mice obtained from the Johns Hopkins University Immune Compromised Animal Core.

### Xenograft models

Mice were injected with cancer cells subcutaneously in 100 μl of PBS (top right flank) for all tumor models. The following cell numbers were used: HT1376 (4 million), UC9 (3 million), UC14 (3 million), RT112 (1.5 million), T24(3 million), and SCaBER (4 million). Mice with tumor volumes of 100 to 200 mm^3^ were used for all experiments. A minimum of four mice were used for all biodistribution studies. A minimum of seven mice were used in PD experiment to account for greater variability.

### PET imaging of mouse xenografts

Mice with tumor volumes of ~100 to 150 mm^3^ were injected with ~200 μCi [7.4 megabecquerels (MBq)] of [^68^Ga]AJ647 in 200 ml of 5% ethanol in saline intravenously and anesthetized under 2.5% isoflurane. PET-computed tomography (CT) images were acquired at 60 min after radiotracer injection at 5 min per bed in an ARGUS small-animal PET/CT scanner (Sedecal, Madrid, Spain) as described previously (*n* = 3, unless otherwise noted). PET-MR images were acquired on a simultaneous 7T Bruker PET-MR scanner. Dynamic imaging was conducted to evaluate the PK of [^68^Ga]AJ647. [^68^Ga]AJ647 was injected (with the help of a catheter) after mice were anesthetized under 2.5% isoflurane on the equipment bed. All PET data were reconstructed using the two-dimensional (2D) ordered subset expectation maximization algorithm and corrected for radioactive decay and dead times. The SUV values were calculated by finding %IA on the basis of a calibration factor obtained from a known radioactive quantity and then normalizing by mice weight. Amide software was used to derive SUV values by drawing the region of interest to closely fit tumors. In particular, MR images were used to identify tumor boundaries manually; these boundaries were transferred to co-registered PET images to derive SUVs. This process was repeated for each mouse individually in a blinded fashion. For the change in accessible Nectin-4 following EV treatment, ΔSUV was calculated by subtracting baseline (pre-treatment) uptake from posttherapy uptake of [^68^Ga]AJ647. TE was quantified by normalizing this change by initial SUV in the same subject controls. Image fusion, visualization, and 3D rendering were accomplished using Amira 2020.3.1 (FEI, Hillsboro, OR).

### Immunohistochemistry

Formalin-fixed, paraffin-embedded tissue sections were baked at 60°C, soaked in xylene to remove paraffin, and then rehydrated through incubations in xylene (1 × 5 min), 100% ethanol (2 × 5 min), 95% ethanol (2 × 5 min), 80% ethanol (2 × 5 min), and H_2_O (1 × 5 min). Antigen retrieval was performed by heating the sections in pH 8.5 ethylenediaminetetraacetic buffer in a decloaking chamber for 20 min. Endogenous peroxide activity was blocked with 3% hydrogen peroxide for 10 min, followed by blocking with 10% goat serum for 1 hour, and then the sections were incubated with a primary anti-human Nectin-4 antibody (polyclonal, no. ab155692, Abcam) at a 1:100 dilution at 4°C overnight. After washing with PBS, the secondary antibody, Signalstain Boost IHC Detection Reagent (horseradish peroxidase), was applied and incubated for 30 min at room temperature. The slides were washed, and immunoreactivity was developed using the ImmPACT DAB substrate. After washing, the slides were counterstained with Mayer’s hematoxylin for 1 min, dehydrated using alcohol and xylene, and then coverslipped. Microscopy was performed by Johns Hopkins University Oncology Tissue Services Core at a 40× resolution. The necrosis region was ignored when selecting fields for immunohistochemistry signal quantification. All analyses were carried out in QuPath version 0.3.0.

### Ex vivo biodistribution

To validate imaging studies, ex vivo biodistribution studies were conducted in mice with tumors of 100 to 200 mm^3^ in size. Mice received ~50 μCi (1.85 MBq) of [^68^Ga]AJ647 in 200 ml of 5% ethanol in saline intravenously and were euthanized at 5 to 180 min after [^68^Ga]AJ647 injection for PK evaluation (*n* = 4 per time point). In all other studies, a 60-min time point was chosen. Selected tissues (blood, skin, muscle, bone, salivary glands, tumor, lungs, heart, liver, pancreas, spleen, kidneys, bladder, and testicles) were collected, weighed, and counted, and their %IA/g values were calculated as described previously (*33*). For EV PD studies, fewer organs were harvested to accommodate a higher number of mice.

### In vitro competition assays

An FITC analog of EV was made using the manufacturer’s recommended protocol (Thermo Fisher Scientific, no. 46409). Briefly, 1 mg of EV was incubated with FITC-NHS ester at a 5:1 molar ratio overnight at 4°C at 0.1 M borate buffer (pH 8.0). Excess unbound FITC-NHS ester was washed off by performing buffer exchange with 1× PBS using three rounds of ultracentrifugation at 4000*g* for 10 min (3-kDa molecular weight cutoff filter, Amicon, no. UFC900308). The effect of nonradioactive AJ647 on EV binding to Nectin-4 was assessed by incubating HT1376 cells at varying concentrations (5.4 μM to 0.01 nM using a serial dilution) of AJ647 for 30 min at 4°C. Unbound AJ647 was washed off using 3 ml of cold PBS. Cells were incubated with EV-FITC for 30 min at 4°C. Excess EV-FITC was washed off using 3 ml of cold PBS. The mean fluorescence intensity was measured using a flow cytometer, and data are reported as the percentage of 0 nM AJ647. For the effect of EV on [^68^Ga]AJ647 binding, EV was used at concentrations of 90 to 0.04 nM using serial dilutions for 30 min at 4°C. Unbound EV was washed off using cold PBS. Then, cells were incubated with 1 μCi of [^68^Ga]AJ647 for an additional 30 min. Excess [^68^Ga]AJ647 was washed off three times with cold PBS. Samples were read in an automated gamma counter, and data are reported as the percentage of 1-μCi standard counts.

### Pilot in vivo experiment to study the EV effect on the tumor uptake of [^68^Ga]AJ647

Male NSG mice were implanted subcutaneously with HT1376 and SCaBER cells (4 million each) on the right and left rostral ends, respectively. On day 25 after cell inoculation (average tumor volume, 200 ± 35 mm^3^), mice were randomized and treated with a single dose of either saline or EV ADC (20 mg/kg) injected intravenously (volume not exceeding 300 μl) or with 200 μl of saline (*n* = 7 per group). Twenty-four hours following the EV dose, [^68^Ga]AJ647 PET scans (*n* = 2 per group) were acquired, and ex vivo biodistribution (*n* = 5 per group) was performed using the protocols mentioned above.

### EV effect on Nectin-4 temporal PD

The dose-time PD of Nectin-4 following EV treatment was studied at doses of 6, 9, and 15 mg/kg. Experiments involving PET imaging were performed first. Male NSG mice were inoculated with 4 million HT1376 cells at day 0. On day 23 (average tumor volume, 150 ± 30 mm^3^), mice were randomized to receive either saline or a single dose of EV (6, 9, or 15 mg/kg) intravenously (*n* = 3 for each dose and *n* = 2 for saline). Mice (*n* = 2) from each dose group and saline group were imaged on days 1, 3, and 6 following EV treatment. Once PET imaging results were analyzed, ex vivo distribution experiment was performed with *n* = 16 to 18 for each dose and *n* = 10 for saline. Treated mice (*n* = 8 or 9 per dose per time point) along with saline controls (*n* = 5 per time point) were euthanized on days 1 and 6. Biodistribution studies were performed at 60 min after [^68^Ga]AJ647 injection, as described above. PD metrics were defined as follows

$$\text{Accessible Nectin}-4={\text{SUV}}_{\text{post}}/{\text{SUV}}_{\text{pre}}*100$$

$$\text{Nectin}-4\;\text{TE}=\left({\text{SUV}}_{\text{pre}}-{\text{SUV}}_{\text{post}}\right)/{\text{SUV}}_{\text{pre}}*100$$

### TE as a noninvasive metric to predict the EV therapy response

Male NSG (*n* = 35) mice were inoculated with 4 million HT1376 cells at day 0. On day 23 (average tumor volume, 170 ± 22 mm^3^), mice were randomized to receive either saline or a single dose of EV (6 or 15 mg/kg) intravenously (*n* = 10 for each group). Mice (*n* = 10) from each dose group were imaged on days 22 (pre-EV) and 29 (6 days post-EV). Saline mice (*n* = 5) were imaged on day 25 to serve as temporal and treatment control. Mouse body weight and tumor measurements were recorded until saline group tumors reached 1500 mm^3^ in average size. Responders and nonresponders were identified using mRECIST for mice published elsewhere (*34*). Briefly, the percentage change in volume relative to the baseline: % tumor volume change (ΔVol_t_) = 100% × [(*V*_t_ − *V*_initial_)/*V*_initial_]. BestResponse was the lowest ΔVol_t_ for *t* ≥ 10 days. For each time *t*, the average ΔVol_t_ from *t* = 0 to *t* was calculated, with the BestAvgResponse defined as the lowest average ΔVol_t_ for *t* ≥ 10 days. mRECIST, adapted from RECIST21, assigned response labels when both the BestResponse and BestAvgResponse conditions are met at the same time. A modified complete response is defined when BestResponse is less than −95% and BestAvgResponse is less than −40%. A modified partial response is assigned when BestResponse is less than −50% and BestAvgResponse is less than −20%. Modified stable disease is defined when BestResponse is less than 35% and BestAvgResponse is less than 30%. Modified progressive disease is assigned when none of these criteria are satisfied.

We defined modified progressive disease as nonresponders (0 response) and stable disease or better as responders (1 response). The TE data and response were fed into Python packages “roc_curve” and “auc” (both from the “sklearn.metrics” library) to generate true positive rates (TPRs) and false positive rates (FPRs) for different TE thresholds. The ROC curve was generated by plotting TPRs (sensitivity) against FPRs (1 − specificity). Optimum TE was the defined threshold at which Youden’s *J* statistic (TPR-FPR) was maximum.

### Statistical analysis

All statistical analyses were performed using Prism 10.0 software (GraphPad Software, La Jolla, CA). Unpaired Student’s *t* test and one-way and two-way analyses of variance (ANOVAs) were used for column, multiple column, and grouped analyses, respectively. *P* values <0.05 were considered statistically significant. Correlation was done using simple linear regression without keeping constant term zero.

### Study approval

All mouse studies were conducted through Johns Hopkins University Animal Care and Use Committee–approved protocols. Protocol M024M175 was approved by B. J. Canning (Chair, Animal Care and Use Committee, 1-877-932-6675).
