# Supplementary material for: Nectin-4 PET for predicting enfortumab vedotin dose-response in urothelial carcinoma
Source: Sci Adv. 2026 Jan 7;12(2):eady1111. doi: 10.1126/sciadv.ady1111 (PMC12778052; doi:10.1126/sciadv.ady1111)
Supplement: Supplementary file 1 — Figs. S1 to S15 Table S1 [file sciadv.ady1111_sm.pdf]

Science Advances
AAAS

Supplementary Materials for

Nectin-4 PET for predicting enfortumab vedotin dose-response in urothelial carcinoma

Akhilesh Mishra *et al.*

Corresponding author: Sridhar Nimmagadda, snimmag1@jhmi.edu

Sci. Adv. **12**, eady1111 (2026)
DOI: 10.1126/sciadv.ady1111

This PDF file includes:

Figs. S1 to S15
Table S1

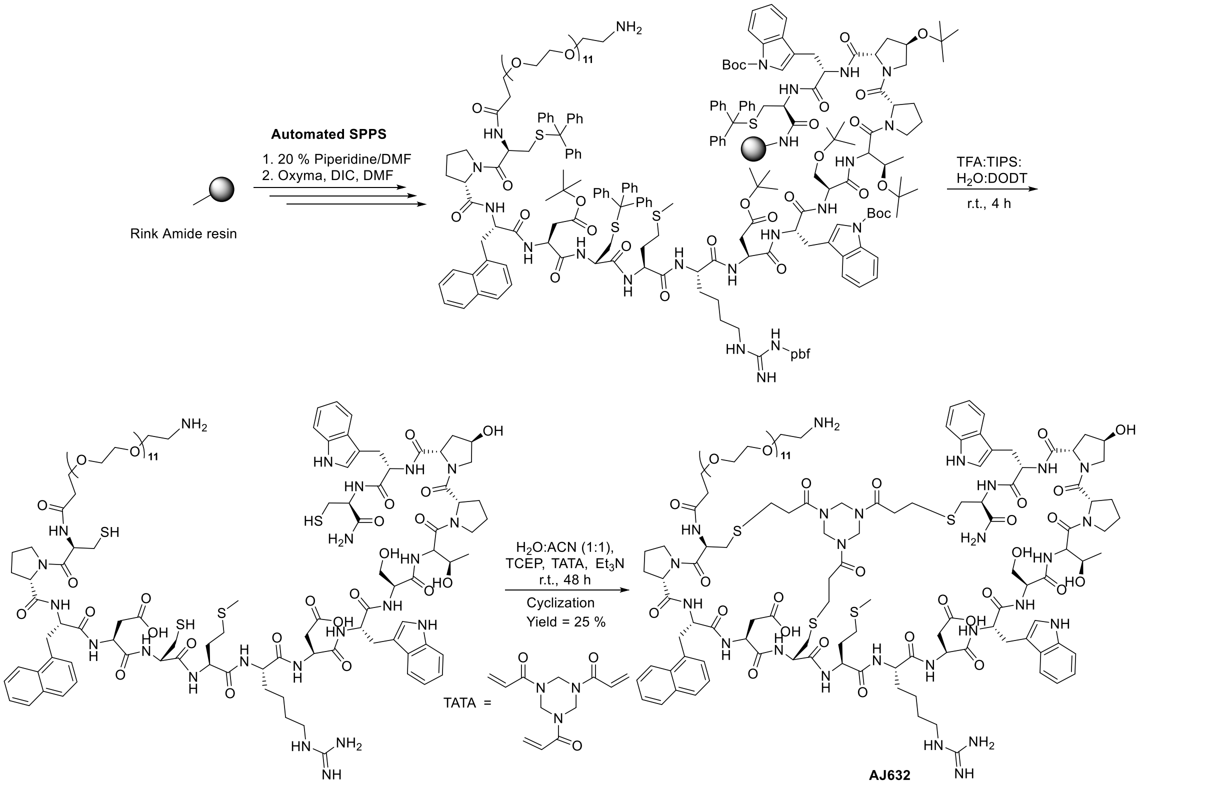

Fig. S1.

The synthesis of AJ632 was achieved through an automated solid-phase peptide synthesis (SPPS) method. First, Fmoc-protected amino acids were sequentially added to Rink amide resin via microwave-assisted coupling reactions. Next, the resulting peptidyl resin underwent treatment with a cleavage cocktail to yield the linear, deprotected peptide. Finally, the linear peptide was cyclized using TATA in the presence of triethylamine (Et3N) in a water: acetonitrile mixture, leading to the formation of AJ632.

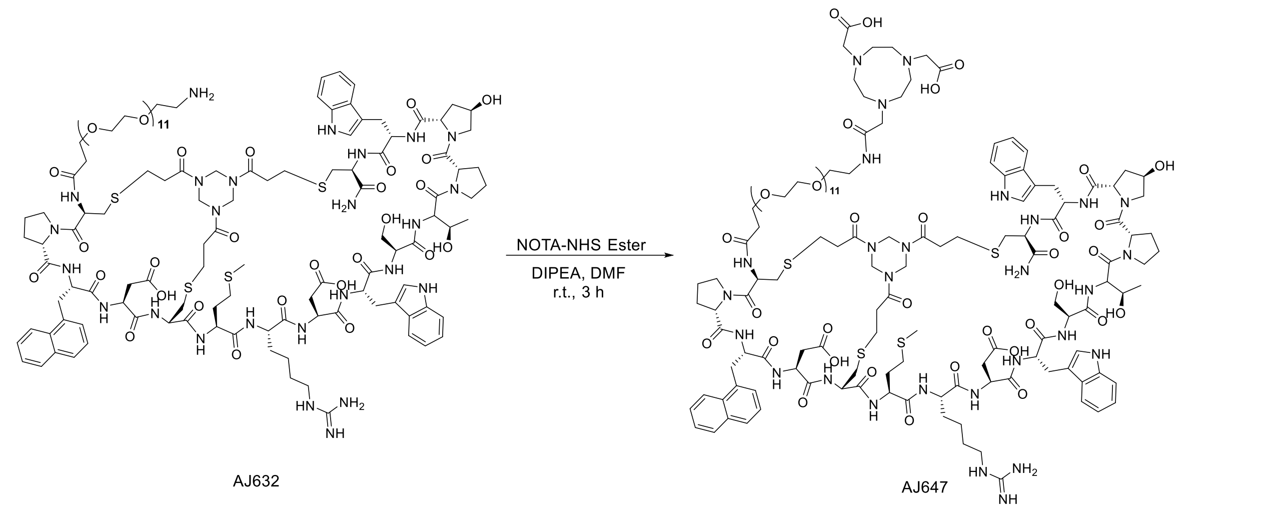

Fig. S2.

Conjugation reaction of AJ632 with NOTA-NHS Ester to Obtain AJ647. The reaction involves adding NOTA-NHS esters, a chelating agent in the AJ632, a peptide, in the presence of diisopropylethylamine (DIPEA) as a base and DMF as a solvent at room temperature for 3 hours. Following the reaction, the product, AJ647, was purified by HPLC.

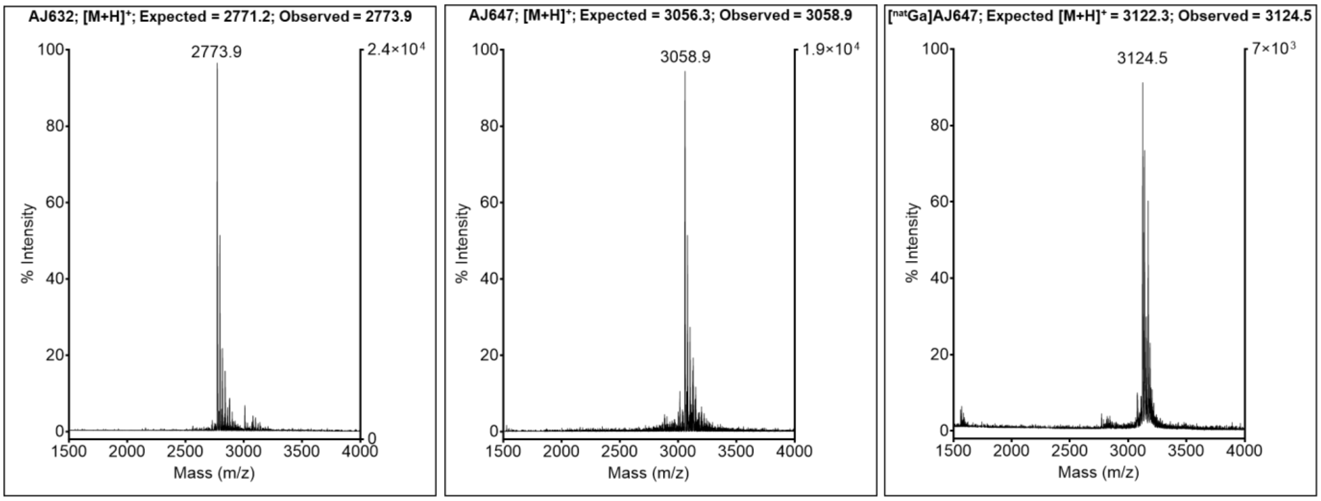

Fig. S3.

Characterization of AJ632, AJ647, and [^{nat}Ga]AJ647 Using MALDI-TOF Mass Spectrometry. This Fig. presents the MALDI-TOF mass spectra for AJ632, AJ647, and [^{nat}Ga]AJ647. Left panel shows the mass spectrum of AJ632 and expected $[M+H]^+$ was 2771.2 and observed $[M+H]^+$ was 2773.9. Middle panel displays the spectrum for AJ647, and expected $[M+H]^+$ was 3056.3 and observed $[M+H]^+$ was 3058.9, indicating successful conjugation of the NOTA chelator to the peptide. Right panel, illustrates the spectrum of [^{nat}Ga]AJ647, and expected $[M+H]^+$ was 3122.3 and observed $[M+H]^+$ was 3124.5, indicating successful incorporation of the non-radiolabeled gallium and showing the structural chemical identity with [^{68}Ga]AJ647.

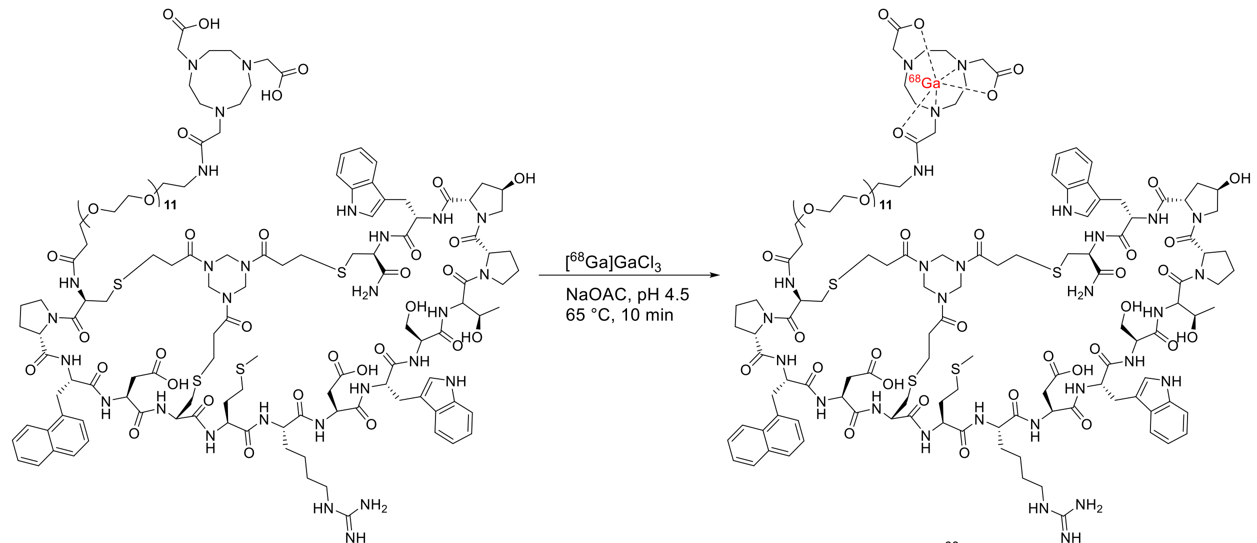

AJ647 [68Ga]AJ647

Fig. S4.

Radiolabeling reaction of AJ647 with [^{68}Ga]GaCl$_3$. This scheme showing the radiolabeling of AJ647 with [^{68}Ga]GaCl$_3$ to produce [^{68}Ga]AJ647. AJ647 was reacted with [^{68}Ga]GaCl$_3$ in a glass vial, where the gallium-68 isotope complexes with the NOTA chelator on AJ647 under optimized heating conditions. Following the radiolabeling reaction, [^{68}Ga]AJ647 was purified by HPLC to remove unreacted [^{68}Ga]GaCl$_3$ and other impurities, yielding the final radiolabeled product for use in in vitro and in vivo studies.

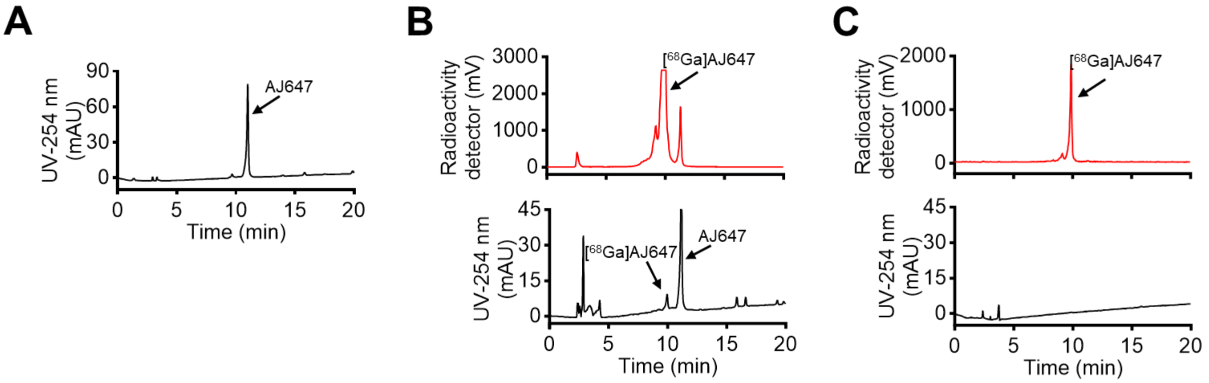

Decay corrected Radiochemical yield (RCY) = 71.2 ± 9.4 % (n = 20)

Radiochemical purity (RCP) = 97 ± 0.8 % (n = 20)

Specific activity = 8-12 GBq/μmol (220-320 mCi/ μmol)

Fig. S5.
Radiolabeling and Characterization of [^{68}Ga]AJ647 A) HPLC chromatograms of AJ647 before radiolabeling. The chromatogram shows the retention time of 11.0 min and >95% of purity of the unlabeled AJ647 peptide, providing a baseline for comparison with the radiolabeled product. **B)** HPLC chromatograms of the purification process following the gallium-68 labeling reaction, showing the separation and purification of [^{68}Ga]AJ647 with the decay-corrected radiochemical yield of 71.2 ± 9.4 (n = 20). **C)** HPLC chromatogram of quality control analysis of the purified radiolabeled product after formulation, showing the purity of >95 % with the molar specific activity of approximately 8-12 GBq/μmol.

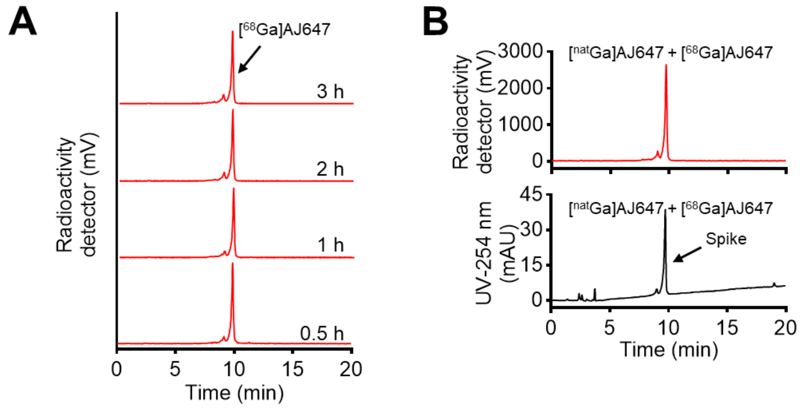

Fig. S6.
Stability of [^{68}Ga]AJ647 A) Stability of [^{68}Ga]AJ647 in formulation buffer over a period of up to 3 hours. This demonstrates the radiotracer's stability and integrity, confirming that [^{68}Ga]AJ647 remains intact and reliable for imaging applications throughout this time frame. **B)** HPLC chromatograms showing the chemical identity of [^{68}Ga]AJ647 with [^{nat}Ga]AJ647. The chromatograms show the retention times and peak profiles of both the radiolabeled and non-radiolabeled forms of AJ647, validating the stable incorporation of gallium-68 and confirming the chemical identity of the radiolabeled product.

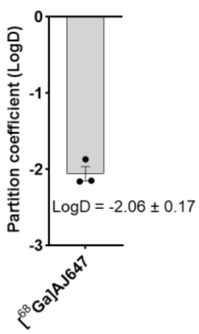

Fig. S7.
Partition coefficient of [^{68}Ga]AJ647 between n-octanol and PBS. The determined Log D value was -2.06 ± 0.17, indicating high aqueous solubility of [^{68}Ga]AJ647.

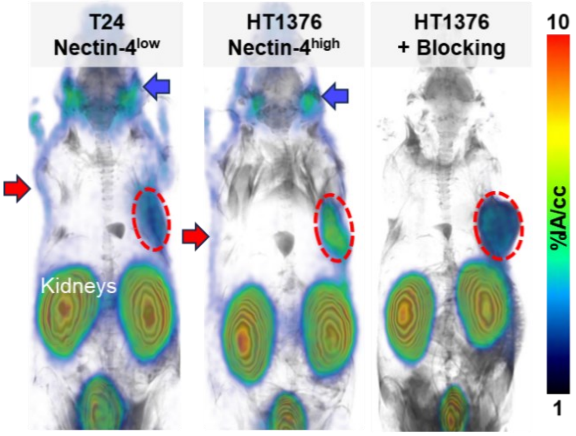

Fig. S8.

[^{68}Ga]AJ647 uptake in tumor xenografts, salivary glands, skin, and Nectin-4 Specificity

Whole-body static PET-MR imaging 60 minutes after injection of approximately 7.4 MBq (~200 μCi) of [^{68}Ga]AJ647 in NSG mice bearing T24 (low Nectin-4; left panel) and HT1376 (high Nectin-4; middle panel) tumors. The images reveal high accumulation of [^{68}Ga]AJ647 in the HT1376 tumor (indicated by a red circle) compared to the T24 tumor. Red arrows points to [^{68}Ga]AJ647 uptake in the skin, and blue arrows indicate accumulation in the salivary glands, demonstrating Nectin-4 dependent uptake in these healthy tissues. To assess specificity, excess non-radioactive AJ647 (2 mg/kg) was pre-injected to block Nectin-4 receptors, followed by whole-body PET-MR imaging (right panel). The resulting image shows reduced radiotracer accumulation in the HT1376 tumor as well as skin and salivary glands, confirming the specificity of [^{68}Ga]AJ647 for Nectin-4.

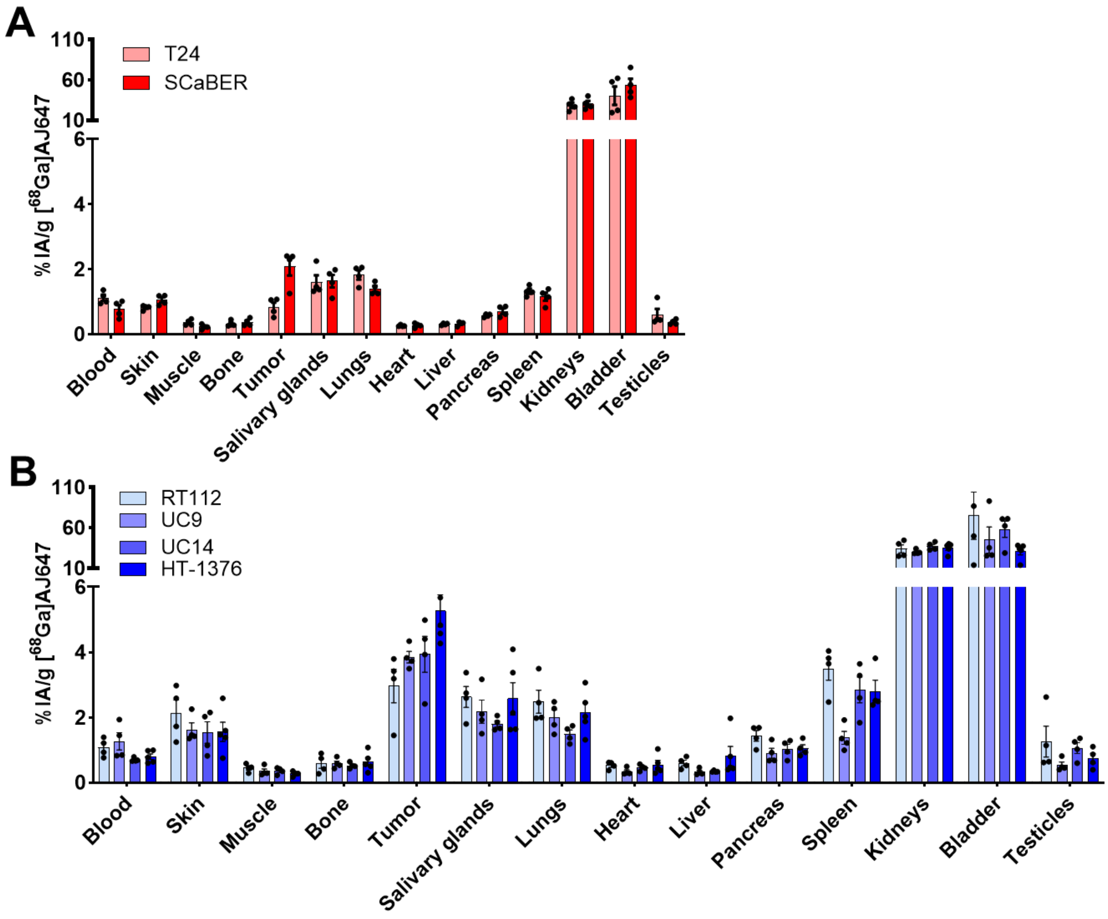

Fig. S9.
Ex vivo biodistribution of [^{68}Ga]AJ647 in NSG mice bearing various bladder cancer xenografts (basal phenotypes in **A**, luminal phonotypes in **B**), as used in Fig. 4. Mice were sacrificed 60 minutes after injection of approximately 740 kBq (~20 μCi) of [^{68}Ga]AJ647. The Fig. shows quantified radiotracer distribution in selected healthy tissues, highlighting the tissue-specific uptake and distribution patterns of [^{68}Ga]AJ647. 2-way ANOVA was used to find differences between the groups and none of the differences were significant (p-value greater than 0.05).

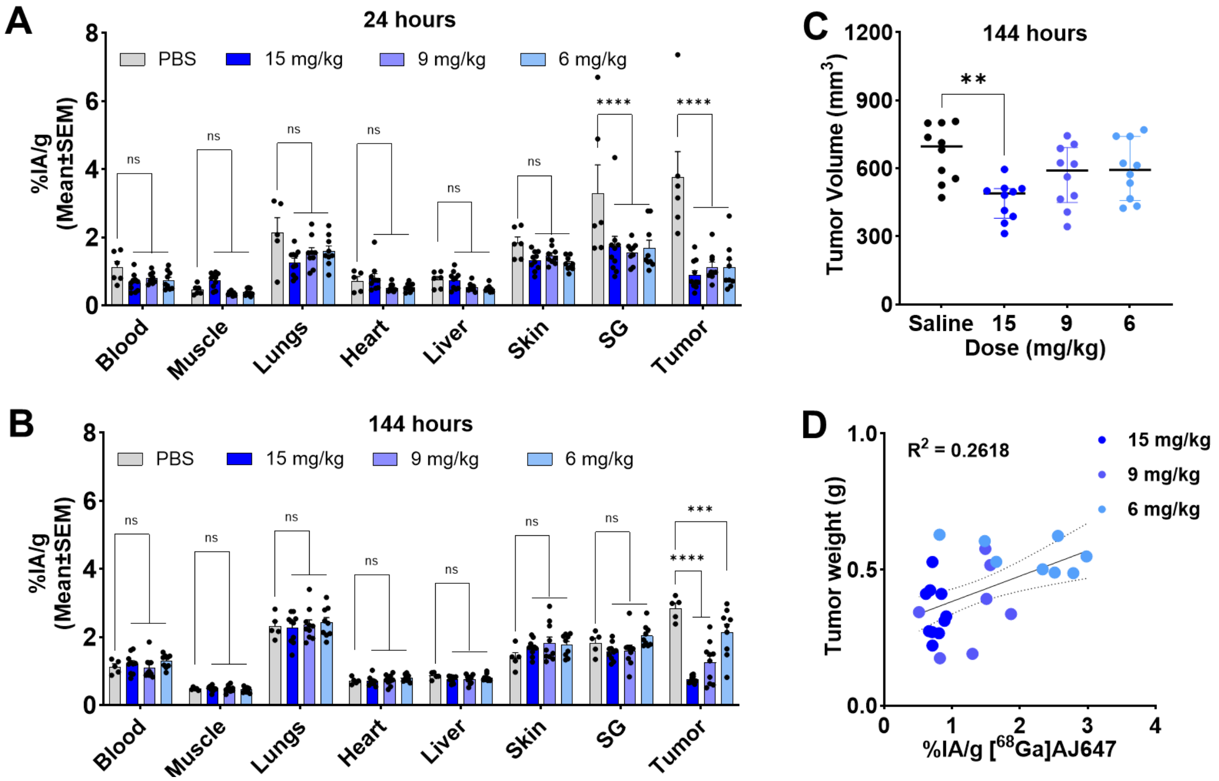

Fig. S10.

Quantification of EV Nectin-4 engagement using [^{68}Ga]AJ647. A and B) Uptake of [^{68}Ga]AJ647 in blood, tumors and tissues of mice bearing HT1376 xenografts used in Fig. 6. All mice were sacrificed at 60 min after injection of ~740 kBq (~20 µCi) [^{68}Ga] AJ647. 2-way ANOVA used to derive p-values. **C)** Final tumor volume of mice used in Fig. 6. **D)** Correlation analysis between Nectin-4 blocking and tumor weight after 6 days of EV administration, indicating a weak correlation, suggesting that [^{68}Ga]AJ647 PET imaging has the potential to predict long-term tumor response to EV treatment. one-way ANOVA used to derive p-values and Pearson correlation used in C.

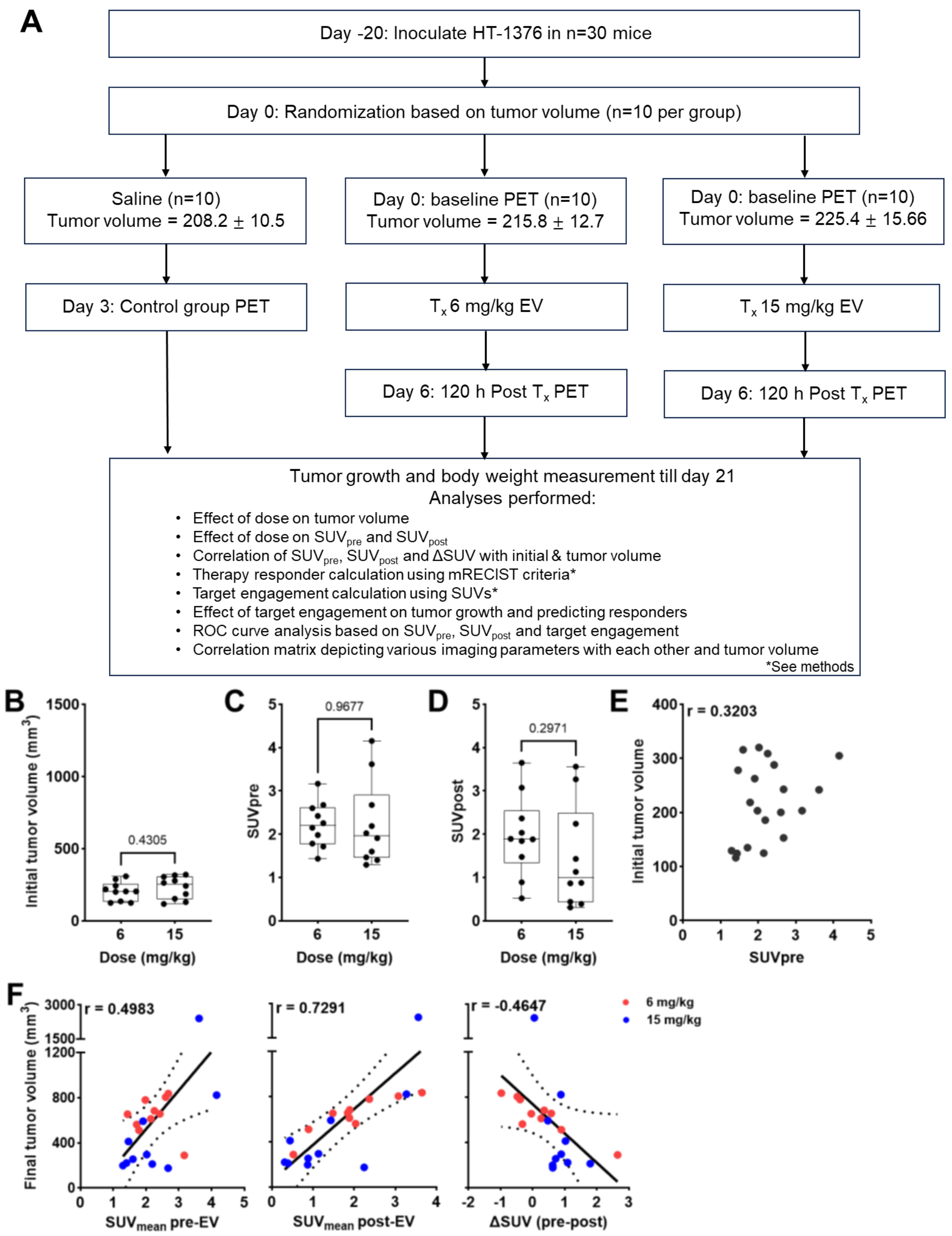

Fig. S11.

Effect of dose on [68Ga]AJ647 uptake. A) Schematic of EV treatment plan imaging studies with **B)** Mice were randomized to have similar tumor volumes before starting the treatment. [68Ga]AJ647 tumor **(C)** SUV_{pre} and **(D)** SUV_{post} shows subtle but not statistically significant differences between the dose groups. **E)** Correlation between initial tumor volume and [68Ga]AJ647 SUV_{pre} for n=20 mice used in the study shown no correlation. **F)** Correlation between final tumor volume and [68Ga]AJ647 SUV (SUV_{Pre}, SUV_{post} and ΔSUV) for mice used in the respective treatment group. Student's t-test used in B-D and Pearson correlation used in E-F.

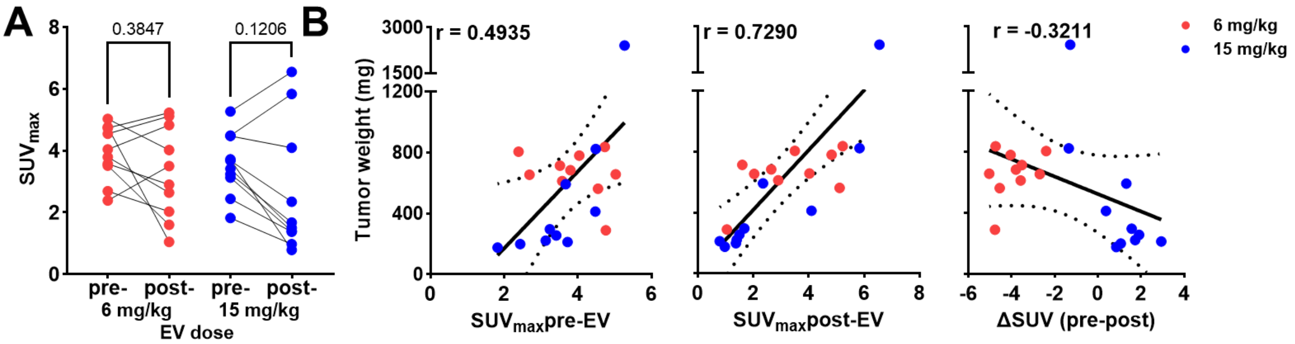

Fig. S12.

Correlates of [^{68}Ga]AJ647 PET SUVmax with final tumor measurements. A) SUV_{max} values for each mouse derived from the PET-MR imaging, comparing uptake before and after EV treatment. **B)** Correlation between tumor weights and SUV_{max} measured before treatment (left), after treatment (middle) and the change in SUVmax (right). Student's t-test used in A (paired) and Spearman correlation used in B.

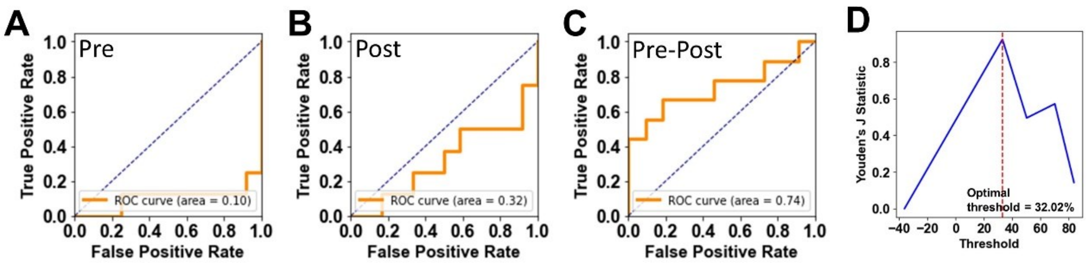

Fig. S13.

Predictive analysis based on different parameters derived from [^{68}Ga]AJ647 PET SUV.

ROC curves based on **A)** SUV_{pre} , **B)** SUV_{post} and **C)** $\Delta SUV_{pre\text{-}post}$ to predict long-term responders.**D)** Youden's J statistic to find optimum threshold to differentiate responders and non-responders.

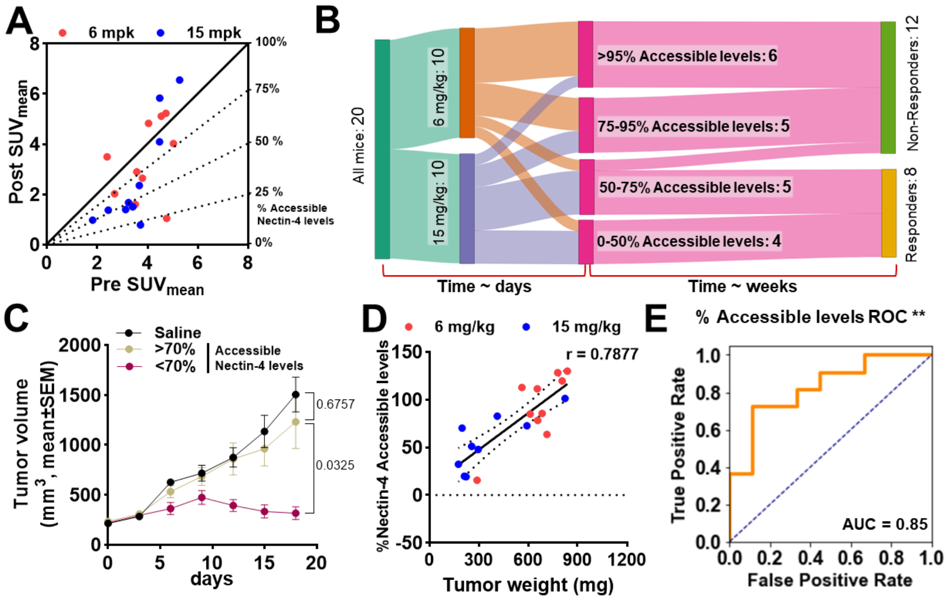

Fig. S14.

[^{68}Ga]AJ647 derived Nectin-4 accessible levels and correlation with EV response. A) Diagonal plot of post-treatment vs baseline SUV, indicating Nectin-4 accessibility. Points farther from the diagonal reflect lower Nectin-4 accessibility after EV treatment, while points above the diagonal indicate higher accessible Nectin-4. **B)** Sankey diagram illustrating the relationship between dose, accessible Nectin-4 levels, and response variability. The left side represents two dosing regimens ("6 mg/kg" and "15 mg/kg," 10 samples each). Data flow into four categories of accessible Nectin-4 levels on the right: <50, 50–75, 75–95, and >95. The width of the connecting bands reflects the number of samples in each transition. Across both regimens, 11 samples exhibited high accessibility (>75), including 6 in >95 and 5 in 75-95% categories. While the 15 mg/kg group showed a dose-dependent shift toward lower accessibility (50–75% and <50%), persistence of high-accessibility samples suggests additional biological or pharmacological factors influencing response beyond dose. **C)** Tumor growth curves stratified by accessible nectin-4 levels, demonstrating growth inhibition in mice with at less than 70% accessible Nectin-4. **D)** Correlation between tumor weights and accessible nectin-4 levels. **E)** ROC curve shows measuring the accessible Nectin-4 is a sensitive predictive measure of response. Pearson correlation was used in E.

	Tumor (pre)	Tumor (post)	Skin (pre)	Skin (post)	Skin Target Engage-ment	SG (pre)	SG (post)	SG Target Engage-ment	Dose	Tumor-to-skin post	Tumor-to-SG post	Tumor Target Engage-ment	Tumor volume
**Tumor (pre)**	1.00	0.67	0.13	-0.08	0.13	0.00	0.16	-0.20	0.01	0.66	0.59	-0.20	0.34
**Tumor (post)**		1.00	0.03	-0.15	0.15	0.11	0.31	-0.30	-0.24	0.99	0.96	-0.82	0.76
**Skin (pre)**			1.00	-0.21	0.74	0.18	0.15	-0.02	0.19	0.06	-0.04	0.06	0.06
**Skin (post)**				1.00	-0.81	-0.20	-0.15	-0.04	0.19	-0.29	-0.09	0.28	-0.36
**Skin Target Engagement**					1.00	0.22	0.18	0.01	-0.01	0.23	0.04	-0.16	0.27
**SG (pre)**						1.00	0.49	0.28	0.00	0.15	-0.02	-0.13	0.44
**SG (post)**							1.00	-0.70	0.14	0.34	0.09	-0.35	0.38
**SG Target Engagement**								1.00	-0.14	-0.29	-0.16	-0.35	-0.11
**Dose**									1.00	-0.25	-0.37	0.48	-0.65
**Tumor-to-skin post**										1.00	0.93	-0.20	0.80
**Tumor-to-SG post**											1.00	0.02	0.71
**Tumor-EV Target engagement**												1.00	-0.84
**Tumor volume**													1.00

Fig. S15.

Correlates of [^{68}Ga]AJ647 PET imaging with final tumor volume. Correlation between different PET imaging derived parameters with final tumor volume. Pearson correlation is used for each pair (n=20).

Ligand	FC	Analyte	Ligand Binding (RU)	MWL (Da)	MWA(Da)	Stochiometric Ratio	R_{max}
Human Nectin-4	2	AJ647	600	44000	3057.5	1:1	41.7
Mouse Nectin-4	4	AJ647	600	45000	3057.5	1:1	40.8

Table S1.
Parameters used in SPR study.
